# Plant *ADH1* promoter acts as an H3K27me3‐associated hyper‐long cold‐responsive promoter

**DOI:** 10.1111/tpj.70248

**Published:** 2025-06-09

**Authors:** Hanako Shimizu, Haruki Nishio, Hiroshi Kudoh

**Affiliations:** ^1^ Center for Ecological Research Kyoto University Hirano 2‐509‐3, Otsu 520‐2113 Japan; ^2^ Data Science and AI Innovation Research Promotion Center Shiga University Banba 1‐1‐1 Hikone 522‐8522 Japan

**Keywords:** week‐scale environmental response, *ADH1*, promoter, H3K27me3, seasonal adaptation, thermal response, *Arabidopsis halleri*

## Abstract

Detecting long‐term environmental trends is a fundamental requirement for organisms living in fluctuating environments to optimize their physiological and developmental responses. This characteristic depends on long‐term memory. However, whether gene promoters alone confer week‐scale environmental responses (WERs) and the mechanisms governing the appearance of these responses at different levels of gene expression—including phenotype, protein, histone modification, and mRNA levels—remains unknown. Herein, we performed a genome‐wide screening for WER promoters using 1‐year‐long time‐series data of a repressive histone modification, H3K27me3, in the promoter region of *Arabidopsis halleri* growing in a natural population. We further analyzed the characteristics of the selected WER promoter using the promoter–reporter lines of *A. thaliana*. H3K27me3 levels in the endogenous *A. halleri ALCOHOL DEHYDROGENASE 1* (*ADH1*) promoter showed WERs, and it responded to 2‐week‐long low temperatures but not to 1‐day‐long low temperatures. Moreover, the fusion of the *ADH1* promoter to the β‐glucuronidase (GUS) reporter gene conferred WER capacity to the GUS protein independently of the mRNA response. The fusion of the coding regions of the *FLOWERING LOCUS C* and *PHYTOCHROME‐INTERACTING FACTOR 4* genes to this promoter successfully modified the WERs of the flowering and petiole elongation phenotypes, respectively, directionally opposite to conventional responses. Overall, these results reveal that the *A. halleri ADH1* promoter alone can confer WERs at the phenotypic, protein, and H3K27me3 levels, and may potentially confer long‐term environmental responsiveness to other genes.

## Significance Statement

A genome‐wide search identified *Arabidopsis halleri ALCOHOL DEHYDROGENASE 1* (*ADH1*) promoter as a responder that exhibits a week‐scale environmental response (WER). In response to cold exposure, the endogenous *ADH1* promoter showed WER in terms of H3K27me3 accumulation. Experimental fusion of this promoter with a reporter gene and representative genes that modify plant morphology confirmed its ability to confer WER at the levels of phenotype, protein, and H3K27me3 accumulation.

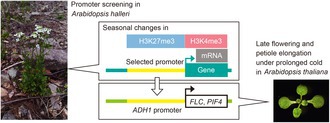

## INTRODUCTION

Plants have specific genes that control their long‐term environmental responses over a week (Kudoh, 2016). The earliest transcriptional response is observed after 15 min or a few hours of exposure to environmental stress, and responses over 24 h after the onset of environmental stimuli are often referred to as long‐term environmental responses (Baier et al., 2019; Oyoshi et al., 2020). In this study, we referred to responses to hyper‐long environmental stresses for over a week, which remain largely unexplored, as week‐scale environmental responses (WERs). Prominent examples of WERs in gene transcription (WER_mRNA) have been reported for key vernalization regulatory genes, such as *VERNALIZATION INSENSITIVE 3* (*VIN3*) and *FLOWERING LOCUS C* (*FLC*) in *Arabidopsis thaliana*, which are upregulated and downregulated, respectively, in response to prolonged cold exposure (Antoniou‐Kourounioti et al., 2018; Hepworth et al., 2018). An analysis of the seasonal dynamics of an *FLC* homologue in a related perennial *Arabidopsis halleri* subsp. *gemmifera* revealed that gene expression is regulated by tracking temperatures over the past 6 weeks (Aikawa et al., 2010).

In the WER of *FLC* gene expression, epigenetic modifications play an important role. Thus, in *FLC*, the levels of H3K27me3, tri‐methylation at the 27th lysine residue of histone H3, are either reduced or accumulated slowly on a timescale of weeks, corresponding to the upregulation and downregulation of gene expression, respectively (Buzas et al., 2011; De Lucia et al., 2008; Nishio, Buzas, et al., 2020; Yang et al., 2017). H3K27me3 exhibits a major silencing mechanism in plants, not only at *FLC* but also at many other loci (Zhang et al., 2007), and the accumulation of H3K27me3 changes slowly in response to seasonal changes in the environment (Nishio, Nagano, et al., 2020). This is in contrast to the representative active histone modification H3K4me3, which accumulates at the transcription start site (TSS) and gene bodies with rapid changes within a day in both *A. halleri* (Nishio, Nagano, et al., 2020) and *A. thaliana* (Sequeira‐Mendes et al., 2014). As H3K27me3 is modifiable on a timescale of weeks but stable on shorter timescales, WER may exist at the transcriptional (WER_mRNA), protein (WER_protein), and histone modification (H3K27me3; hereafter WER_H3K27me3) levels.

Gene promoters exhibiting seasonal H3K27me3 changes, independent of genic regions, confer WERs by controlling gene function. Such a promoter, if available, could aid in elucidating the roles of H3K27me3 in long‐term environmental responses. However, seasonal H3K27me3 changes at promoters are rare in plants because H3K27me3 is mostly deposited in genic regions (Nishio, Nagano, et al., 2020; Zhang et al., 2007). In *FLC*, for example, H3K27me3 accumulates mainly at the TSS and gene body regions in response to long‐term cold exposure (Buzas et al., 2011; Yang et al., 2017).

**Figure 1:**
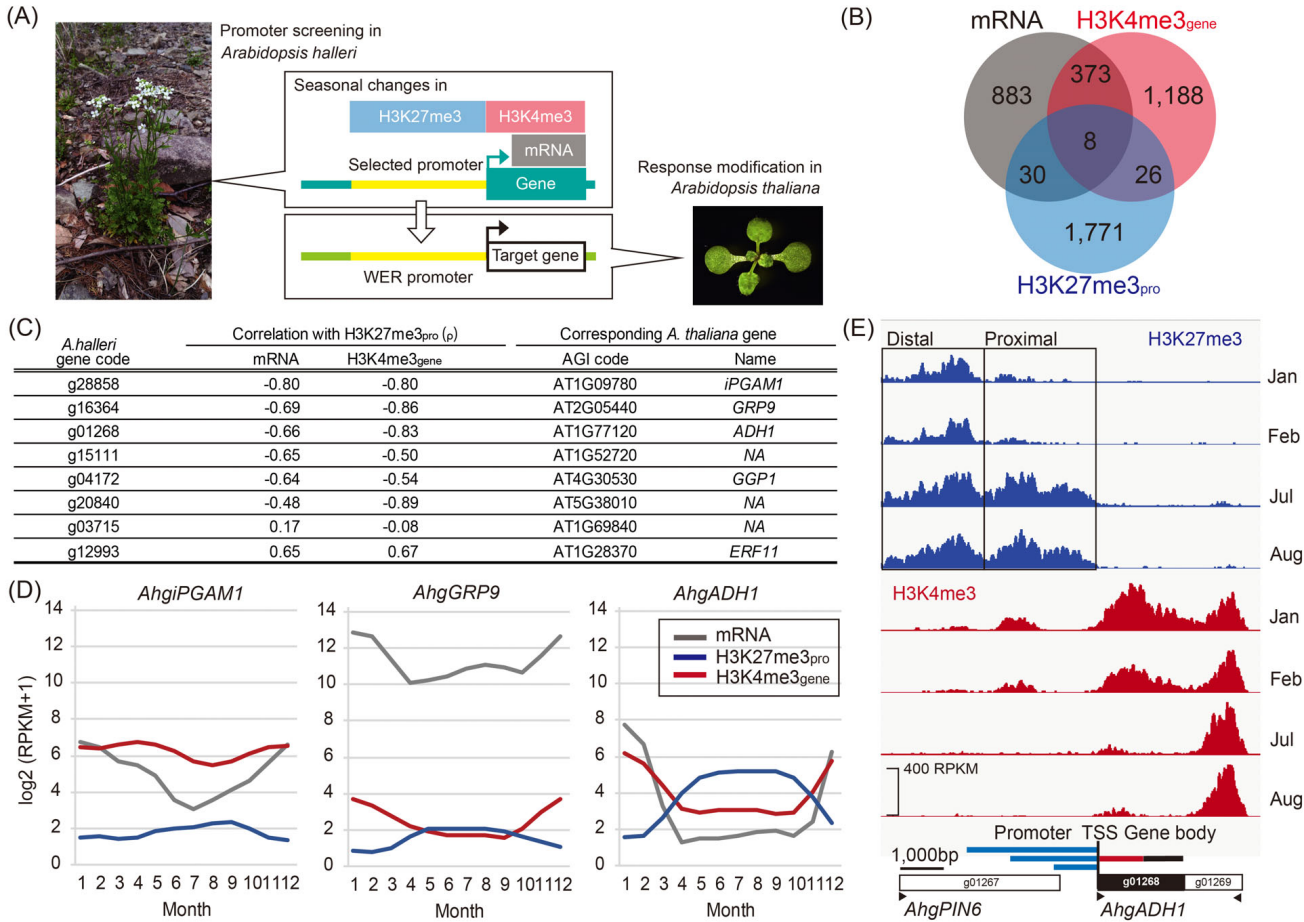
Screening of week‐scale environmental response (WER) promoters using the seasonal transcriptome and histone modification data of *Arabidopsis halleri* in its natural habitat. (A) Schematic depicting the research strategy adopted to identify and apply the WER promoters of *A. halleri* and *A. thaliana*. (B) Venn diagram showing eight *A. halleri* genes with seasonal changes in mRNA, H3K4me3_gene_, and H3K27me3_pro_ levels. (C) Eight genes are listed in ascending order of Spearman's rank correlation coefficient (*ρ*) between H3K27me3_pro_ and mRNA. The *ρ* values between H3K27me3_pro_ and H3K4me3_gene_ and corresponding *A. thaliana* genes are also shown. (D) Monthly changes in the mRNA, H3K27me3_pro_, and H3K4me3_gene_ levels of the top three genes. H3K27me3_pro_ and H3K4me3_gene_ levels in (B–D) are calculated for the 2 kb upstream and 1 kb downstream regions from the transcription start site (TSS), respectively. (E) Accumulation of H3K27me3 and H3K4me3 in the *A. halleri ALCOHOL DEHYDROGENASE 1* (*AhgADH1*) gene and adjacent regions in winter (January and February) and summer (July and August). At the bottom, positions of the *AhgADH1* gene (black box), TSS (vertical black line), regions 3, 2, and 1 kb upstream (upper, middle, and lower blue lines, respectively) and 1 kb downstream of the TSS (red line), gene body (red line + horizontal black line), and upstream and downstream genes (white boxes) are shown. Analyses in (B–E) were performed using the seasonal data of *A. halleri* in its natural habitat (Nishio, Nagano, et al., 2020).

In this study, we used a genome‐wide screening approach to identify rare promoters showing seasonal differences in H3K27me3 accumulation only in the promoter regions (Figure 1A). Additionally, we examined whether the selected promoter conferred WERs at the phenotype, protein, H3K27me3, and mRNA levels. The screening approach is unique, as it uses existing H3K27me3 chromatin immunoprecipitation (ChIP)‐seq data collected monthly for a year in a natural population of *A. halleri* (Nishio, Nagano, et al., 2020). While this dataset reports clear seasonal changes in H3K27me3 accumulation in the genic regions of 1804 genes (Nishio, Nagano, et al., 2020), no such analysis has been performed in the promoter regions.

In this study, screening identified a relatively well‐known promoter involved in the environmental response, the *ALCOHOL DEHYDROGENASE 1* (*ADH1*) promoter, as the first candidate WER promoter. Plant *ADH1* confers both abiotic and biotic stress resistance (Shi et al., 2017), and several *cis*‐motifs in this promoter respond to short‐term stresses, such as exposure to cold (Dolferus, Jacobs, et al., 1994; Kusano et al., 1995), abscisic acid (De Bruxelles et al., 1996; Dolferus, De Bruxelles, et al., 1994; Guiltinan et al., 1990; Macnicol & Jacobsen, 2001), and hypoxia (Gasch et al., 2016; Hoeren et al., 1998; Paul & Ferl, 1991). A 1 kilobase (kb) promoter fragment (−964 to +53) is sufficient to confer short‐term stress responsiveness and tissue‐specific expression patterns in *A. thaliana ADH1* (hereafter *AtADH1*) (Dolferus, Jacobs, et al., 1994). Although the *ADH1* promoter is among the most studied plant promoters, our screening revealed a previously unreported characteristic of the *A. halleri ADH1* (*AhgADH1*) promoter, WER, which may involve changes in H3K27me3 levels. We further analyzed the characteristics of the *AhgADH1* promoter as a WER promoter by establishing a series of *A. thaliana* promoter–reporter lines (Figure 1A). We also assessed whether the *AhgADH1* promoter confers long‐term environmental responsiveness to specific plant genes (Figure 1A).

## RESULTS

### *AhgADH1* is a putative H3K27me3‐associated WER promoter

To identify the candidate H3K27me3‐associated WER promoters, we used seasonal time‐series transcriptome and genome‐wide H3K4me3 and H3K27me3 data from *A. halleri* (Nishio, Nagano, et al., 2020). Eight genes showed seasonality in gene expression (mRNA) related to changes in H3K4me3 levels within a 1 kb region downstream of the TSS (H3K4me3_gene_) and H3K27me3 levels within a 2 kb region upstream of the TSS (H3K27me3_pro_; Figure 1A,B). The top three genes with the highest negative time‐series correlations between their H3K27me3_pro_ levels and mRNA expression were the homologues of *2,3‐BIPHOSPHOGLYCERATE‐INDEPENDENT PHOSPHOGLYCERATE MUTASE 1*, *GLYCINE‐RICH PROTEIN 9*, and *ADH1* (Figure 1C). Moreover, these genes also showed strong negative correlations between their H3K27me3_pro_ and H3K4me3_gene_ levels (Figure 1C). We selected *AhgADH1* for further analysis based on two criteria: (1) it showed the most pronounced seasonality in H3K27me3_pro_ (Figure 1D; Figure S1A) (2) it was the only gene repeatedly selected as a candidate when we designated the promoter region as either 1 or 3 kb upstream of the TSS instead of 2 kb. As the expression levels of *AhgADH1* were upregulated in winter and downregulated in summer and its H3K27me3_pro_ levels showed a high negative correlation with mRNA and H3K4me3_gene_ levels (Figure 1D,E; Figure S2), we hypothesized that the *AhgADH1* promoter exhibits a WER to prolonged cold conditions.

**Figure 2:**
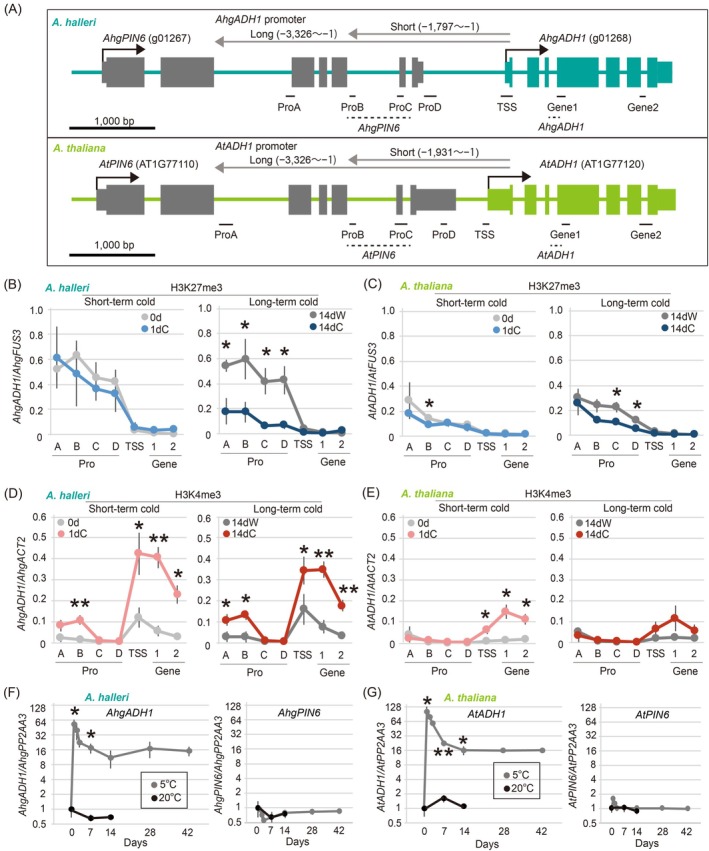
Long‐term cold responses of endogenous *AhgADH1* and *AtADH1*. (A) Gene structures of *AhgADH1* and *AtADH1* (upper and lower diagrams, respectively). Gray arrows denote the regions considered as *ADH1* promoter +5′‐untranslated region (UTR). Boxes represent the exons and UTRs (thinner boxes), and colored and gray boxes correspond to those for *ADH1* and *PIN6* (an overlapping gene with putative *ADH1* promoter). Colored horizontal lines represent the introns and intergenic sequences. Black solid and dotted lines indicate the positions of the amplicons used for chromatin immunoprecipitation (ChIP)–quantitative polymerase chain reaction (qPCR) (B–E) and reverse transcription (RT)‐qPCR (F, G), respectively. (B–E) Levels of H3K27me3 (B, C) and H3K4me3 (D, E) accumulation at different regions of *AhgADH1* (B, D) and *AtADH1* (C, E) in response to short‐term cold exposure (0d and 1dC: before and 1‐day‐long cold exposure, respectively; left diagram) and long‐term warm and cold exposure (14dW and 14dC: 14‐day warm and cold exposure, respectively; right diagram) quantified via ChIP‐qPCR. H3K27me3 and H3K4me3 levels were calculated relative to the levels at *FUS3* and *ACT2* homologues, respectively, in each species. Asterisks indicate significant differences (*P < 0.01; **P < 0.001; Welch's *t*‐test, two‐sided) between 0d and 1dC (B, D) or 14dW and 14dC (C, E). (F, G) Time‐series changes in *ADH1* and *PIN6* gene expression levels quantified via RT‐qPCR (left and right diagrams, respectively) in *A. halleri* (F) and *A. thaliana* (G) under 5°C (for 42 days, gray lines) and 20°C (for 14 days, black lines) conditions. Expression levels were calculated relative to those of the *PP2AA3* homologue in each species, with the value at 0d = 1. Asterisks indicate the significant differences between 0d and 1dC, 7dW and 7dC, and 14dW and 14dC (*P < 0.01; **P < 0.001; Welch's *t*‐test, two‐sided). In (B–G), mean ± standard deviation (*n* = 4) values are shown. qPCR primers are listed in Table S1.

The H3K27me3‐enriched region in the *AhgADH1* promoter largely overlapped with a neighboring upstream gene, a homologue of *PIN‐FORMED 6* (*AhgPIN6*; Figure 1E; Figure S1B). Two regions of the *AhgADH1* promoter showed seasonal changes in H3K27me3 levels, and reduction in H3K27me3 levels was more pronounced in the proximal region than in the distal region (Figure 1E). The proximal region overlapped with a part of the second intron and downstream of *AhgPIN6*, and the distal region overlapped with the second exon and upstream of *AhgPIN6* (Figure 1E). The mRNA expression of *AhgPIN6* and H3K4me3 levels in its TSS region were consistently low throughout the year (Figure S3). Furthermore, changes in H3K27me3 at the *AhgPIN6* gene body were not associated with either its own mRNA or H3K4me3 levels (Figure S3) but showed strong negative associations with *AhgADH* mRNA and *AhgADH1* H3K4me3. Therefore, we hypothesized that the 3′ half of the *AhgPIN6* gene body functions as the *AhgADH1* promoter in long‐term cold response.

Four of the eight initially selected genes, including *AhgADH1*, overlapped with the upstream gene within 2 kb of the TSS (Figure S1B). This frequency (4/8) was not significantly different from that for all genes (15 972/ 32 367, *P*‐value >0.9999 in Fisher's exact test, Figure S1C); therefore, the overlap with neighboring upstream genes can be explained by chance. We further analyzed the presence of common sequence motifs shared by the promoters (2 kb from the TSS) of the eight genes using XSTREAM (https://meme‐suite.org/meme/tools/xstreme). The promoters of all eight genes shared a motif for binding to ARABIDOPSIS THALIANA HOMEOBOX proteins (Figure S1D); however, this motif was found in the promoters of 42.7% (19 493 genes) of genes in *A. halleri*. In the promoters of the top three genes (Figure 1C), we found three shared motifs that had not been characterized and should be the subject of future analysis (Figure S1E).

### H3K27me3 levels in endogenous *AhgADH1* and *AtADH1* promoters exhibit WERs

As H3K27me3 removal and *AhgADH1* activation occur in winter, we tested whether the *ADH1* promoter satisfied the requirements of the WER promoter by examining how long‐term cold treatment affects histone modification levels and expression of *ADH1*. We analyzed the promoter region of *AtADH1* in addition to that of *AhgADH1* because we used *A. thaliana* for further gene recombination experiments. We observed that the structure of the *AhgADH1* gene was similar to that of *AtADH1* with 95.4 and 88.0% nucleotide sequence similarity in their coding and gene body regions, respectively (Figure 2A). For both *AhgADH1* and *AtADH1*, their upstream neighboring genes *AhgPIN6* and *AtPIN6*, respectively, were located close to them (Figure 2A). For the 3326 bp upstream of the TSS of *AhgADH1* and *AtADH1* (to the 2nd intron of *AhgPIN6*), the similarity was 78.5%. We designed amplicons along the promoter and gene body regions of *AhgADH1* and *AtADH1* and examined H3K27me3 accumulation using ChIP–quantitative polymerase chain reaction (ChIP‐qPCR) (Figure 2A; Figure S4A). H3K27me3 accumulated at the *AhgADH1* promoter before treatment (Figure 2B, left) and remained high under warm conditions for 14 days (Figure 2B, right). Exposure to cold for 1 day did not significantly alter H3K27me3 (Figure 2B, left), whereas cold for 14 days largely eliminated H3K27me3 (Figure 2B, right). Thus, H3K27me3 levels in the *AhgADH1* promoter showed a clear response to prolonged cold exposure (Figure 2B). In *AtADH1*, H3K27me3 levels were low at the pre‐treatment stage and remained low under exposure to cold conditions for both 1 and 14 days treatment periods, whereas H3K27me3 accumulated in response to 14 days of prolonged exposure to warmth (Figure 2C). Moreover, H3K27me3 accumulation in the *ADH1* gene body was consistently low throughout the experiment in both species (Figure 2B,C). Although the initial levels of H3K27me3 before the temperature treatment were high and low at the *AhgADH1* and *AtADH1* promoters, respectively, reflecting the age of the plants used for the experiments (28‐day old and 14‐day old in *A. halleri* and *A. thaliana*, respectively, grown under warm), 14‐day cold and 14‐day warm resulted in low and high accumulation of H3K27me3, respectively, for both species. Therefore, these results showed that H3K27me3 at the *ADH1* promoter in both *A. halleri* and *A. thaliana* had a WER to the over‐week thermal conditions.

We investigated whether WER is associated with H3K4me3 accumulation and gene expression. In contrast to the results observed for H3K27me3, H3K4me3 (TSS and gene body) did not show WER and accumulated in response to the 1‐ and 14‐day cold treatments (Figure 2D,E). In addition to H3K4me3 accumulation in the TSS and gene body regions of *ADH1*, we observed H3K4me3 accumulation in regions A and B of the *AhgADH1* promoter in response to the cold treatments (Figure 2D) and minor H3K4me3 accumulation in region A of the *AtADH1* promoter without the cold treatments (Figure 2E). At the gene expression level, *ADH1* was upregulated by 1‐day‐long exposure to cold stress and remained at high levels during prolonged exposure to cold stress (42 days) in both species (Figure 2F,G, left). In both *A. halleri* and *A. thaliana*, the endogenous expression levels of *PIN6* were low throughout the experiment (Figure 2F,G, right).

### *AhgADH1* promoter confers WER capacity to β‐glucuronidase (GUS) in response to prolonged cold stress

**Figure 3:**
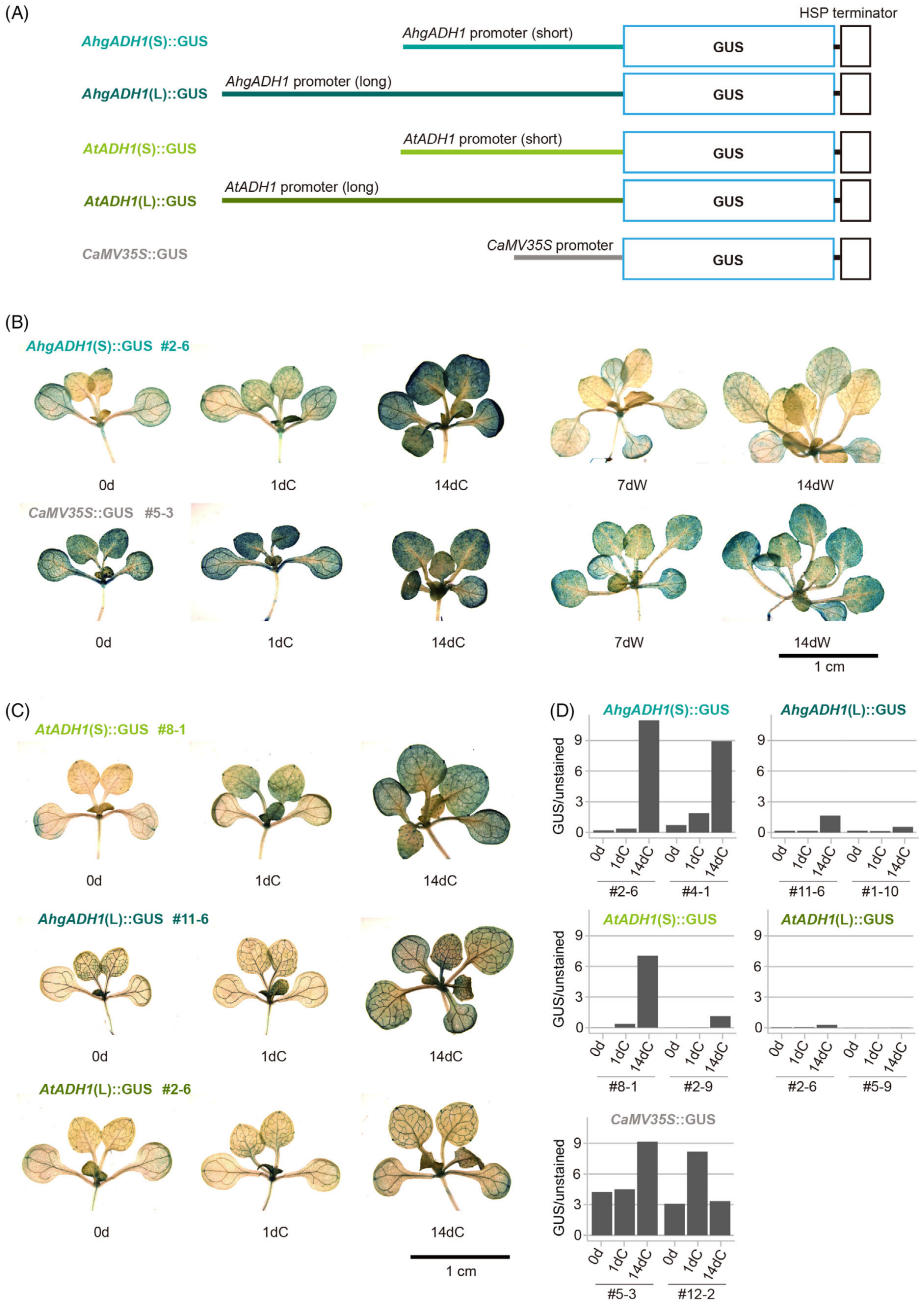
Evaluation of a series of *ADH1* promoters based on GUS signaling in response to short (1 day) and long (14 days) cold exposure. (A) Schematic diagram of five promoter–reporter constructs [*AtADH1* (L), *AtADH1* (S), *AhgADH1* (L), *AhgADH1* (L), and *CaMV35S*]. Colored horizontal lines, colored open boxes, and black open boxes indicate the promoters, coding sequences, and heat shock protein (HSP) terminator, respectively. (B, C) Images of transformed *A. thaliana* lines with different constructs exposed to both cold and warm conditions (B) and exclusively cold conditions (C). For each of the five construct types, representative results from the first lines are shown. Images of the aerial parts of seedlings are also shown. Scale bar in (B, C) represents 1 cm. (D) Quantification of GUS signals in the first and second representative lines, expressed as the ratios of GUS‐stained to unstained tissue areas.

To determine whether the *ADH1* promoter confers the WER_protein in the transgenic lines, we introduced promoter–GUS constructs into *A. thaliana* and examined GUS expression in response to prolonged cold stress. Two sets of *ADH1* promoters (long and short regions) from *A. halleri* and *A. thaliana*, *AhgADH1* (L) and *AhgADH1* (S) and *AtADH1* (L) and *AtADH1* (S) (Figures 2A and 3A), respectively, were tested. They were 3326, 1797, 3326, and 1931 bp upstream from the 5′‐ end of the coding region, respectively. Long promoters contain up to the 2nd intron, whereas short promoters contain up to the 5th intron of *PIN6* (Figure 2A). These four promoter fragments were fused to the 5′‐side of the coding region of the *GUS* gene (Figure 3A). The cauliflower mosaic virus 35S (*CaMV35S*) promoter–*GUS* construct, which induced GUS expression irrespective of temperature, was used as a control (Figure 3A). We evaluated the WERs of the promoters by examining the GUS signals in the aerial parts at 0 (before) and 1 and 14 days after exposure to cold (0d, 1dC, and 14dC, respectively) and warm conditions (7dW, and 14dW, respectively) (Figure 3B–D; Figure S4A). In this experiment, GUS staining increased in response to prolonged exposure to cold conditions (0d = 1dC <14dC in Figure 3B, upper), whereas GUS staining remained weak in response to prolonged exposure to warm conditions (0d = 7dW = 14dW in Figure 3B, upper). Therefore, the strong GUS staining at 14dC can be interpreted as a WER rather than a developmental effect. The *AhgADH1* (S) and *AtADH1* (S) promoters showed clear WERs, and most lines showed enhanced GUS signals at 14dC than at 0d and 1dC (Figure 3B,C). GUS signals at 14dC were stronger in *AhgADH1* (S)::*GUS* lines than in *AtADH1* (S)::*GUS* (Figure 3B–D). *CaMV35S*::*GUS* lines did not show increased GUS signals during the experiment, suggesting that the accumulation of the GUS protein in these lines was negligible under cold conditions (Figure 3B, lower). *AhgADH1* (L)::*GUS* and *AtADH1* (L)::*GUS* lines showed vasculature‐specific GUS signals with no or weak responses to cold stress (Figure 3C), indicating that regions specific to the *AhgADH1* (L) and *AtADH1* (L) promoters conferred tissue specificity. Overall, these results suggest that the *AhgADH1* (S) promoter is the potential H3K27me3‐associated WER_protein promoter, at least in the transgenic lines.

### *AhgADH1* (S):: *GUS* Line exhibits WER_H3K27me3 at the genic region

**Figure 4:**
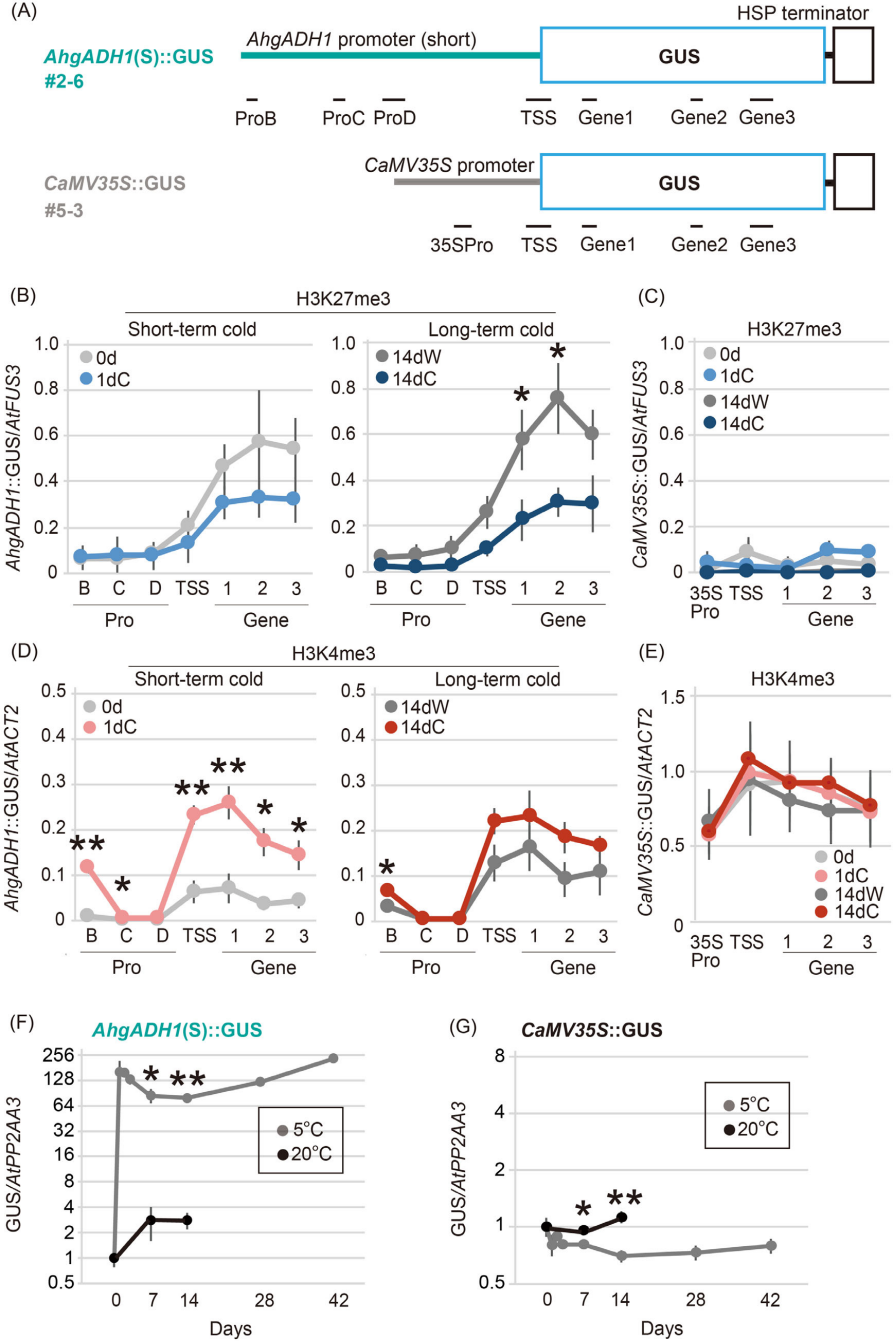
Responses of H3K27me3, H3K4me3, and gene expression levels to varying durations of cold exposure at the transformed WER promoter–GUS locus. (A) Schematic diagrams of *AhgADH1* (S)::*GUS* and *CaMV35S*::*GUS* lines used as the WER and overexpression lines, respectively. The descriptions of lines and boxes are provided in the legend of Figure 3A. Solid black lines indicate the positions of the amplicons used for ChIP‐qPCR (B–E). (B–E) Levels of H3K27me3 (B, C) and H3K4me3 (D, E) accumulation in the *AhgADH1*(S)::*GUS* (B, D) and *CaMV35S*::*GUS* (C, E) lines in response to short‐term cold exposure (0d and 1dC: before and 1‐day cold exposure, respectively; left diagram) and long‐term warm and cold exposure (14dW and 14dC: 14‐day warm and cold exposure, respectively; right diagram) quantified via ChIP‐qPCR. ChIP‐qPCR amplicons at *AhgADH1* (S) are designed to amplify only the introduced promoter, not the endogenous *AtADH1* promoter. H3K27me3 and H3K4me3 levels were calculated relative to the levels at *AtFUS3* and *AtACT2*, respectively. For H3K27me3 levels in the *CaMV35S*::*GUS* line, the values were set to 0.0001 if they were below the detectable levels. Asterisks indicate the significant differences (*P < 0.01; **P < 0.001; Welch's *t*‐test, two‐sided) between 0d and 1dC or 14dW and 14dC. (F, G) Time‐series changes in the expression levels of the *GUS* gene under cold (5°C for 42 days, gray lines) and warm (20°C for 14 days, black lines) conditions in *AhgADH1* (S)::*GUS* (F) and *CaMV35S*::*GUS* (G) lines quantified via RT‐qPCR. Expression levels were calculated relative to those of *AtPP2AA3*, with the mean value at 0d = 1. Asterisks indicate the significant differences between 0d and 1dC, 7dW and 7dC, and 14dW and 14dC (*P < 0.01; **P < 0.001; Welch's *t*‐test, two‐sided). In (B–G), mean ± SD (*n* = 4) values are shown. All qPCR primers are listed in Table S1.

As the *AhgADH1* (S)::*GUS* line showed WER protein (GUS activity), we investigated whether WERs were observed to H3K27me3, H3K4me3, and gene expression levels. For this, we developed ChIP‐qPCR amplicons unique to *AhgADH1* (S) to distinguish exogenous *AhgADH1* promoter, which was derived from a different species, from endogenous *AtADH1* promoters for the H3K27me3 and H3K4me3 analyses (Figure 4A). In *AhgADH1* (S)::*GUS*, H3K27me3 levels exhibited WER, and H3K27me3 accumulation was significantly lower in response to the cold treatments than after exposure to warm conditions for 14 days (Figure 4B, right). The H3K27me3 levels at 1dC and 14dC were similar, and the WER_H3K27me3 occurred as a difference in the accumulation level in the warm (Figure 4B). Although we originally screened the *ADH1* promoter for H3K27me3 accumulation, the promoter in *AhgADH1* (S)::*GUS* caused H3K27me3 accumulation and significant WER at the gene body of the *GUS* locus (Figure 4B). H3K27me3 accumulation was negligible in *CaMV35S*::*GUS* lines under all conditions (Figure 4C). In contrast to WER_H3K27me3, H3K4me3 levels in the gene body and B regions of the promoter increased within a day in response to the cold treatments (Figure 4D). H3K4me3 accumulation in *CaMV35S*::*GUS* was consistently high and did not respond to the cold treatments (Figure 4E). In *AhgADH1* (S)::*GUS*, the expression levels of the *GUS* gene increased rapidly within 1 day in response to cold stress and remained at high levels during prolonged cold exposure (Figure 4F). The expression levels of the gene remained low under warm conditions until 14dW (Figure 4F). This expression pattern of the *GUS* gene was replicated in an additional *AhgADH1* (S)::*GUS* line and was similar to that of endogenous *AhgADH1* (Figure 2F, compared side by side in Figure S5). In *CaMV35S*::*GUS*, the expression levels of the gene remained constant regardless of temperature (Figure 4G). Overall, in *AhgADH1* (S)::*GUS*, changes in GUS protein expression and H3K27me3 accumulation exhibited WERs, whereas those in *GUS* gene expression and H3K4me3 levels exhibited short‐term environmental responses.

### *AhgADH1* (S) promoter is necessary and sufficient for WER_protein

To confirm that the *AhgADH1* (S) promoter is necessary for the WER, we cloned the coding region of the enhanced green fluorescent protein (EGFP) downstream of *AhgADH1* (S) and shorter promoters and generated transgenic lines (*A. thaliana*) bearing *AhgADH1* (S)::*EGFP*, *AhgADH1*(660)::*EGFP*, and *AhgADH1*(558)::*EGFP* constructs. The promoter regions used in the latter two constructs were 660 and 558 bp upstream from the 5′‐ end of the coding region of *AhgADH1*, respectively, and did not overlap with the *AhgPIN6* gene body (Figure S6). All three compounds contained cold‐responsive elements, as previously reported (Figure S6; Dolferus, Jacobs, et al., 1994). We observed EGFP fluorescence and measured EGFP expression at 0d, 1dC, and 14dC (Figure S4A). EGFP signals were observed only in *AhgADH1* (S)::*EGFP* at 14dC (Figure S7A). In the reverse transcription‐qPCR (RT‐qPCR) data, EGFP expression levels were upregulated in all lines at 1dC except for a single line; however, significant upregulation was maintained until 14dC only in *AhgADH1* (S)::*EGFP* (Figure S7B). These results indicate that the *AhgADH1* (S) promoter is not only sufficient but also necessary for conferring WER _protein to cold stress.

### WER of the *AhgADH1* (S) promoter shows memory and priming effects

To evaluate whether the WER of *AhgADH1*(S) promoter exhibited memory and priming effects after the cold treatments, we treated the plants with warm breaks after 1 and 14 days of cold treatments (1dC and14dC, respectively, in Figure S4B). We evaluated the changes in the expression of EGFP in the *AhgADH1*(S)::EGFP line and that of *AhgADH1* in *A. halleri* in response to the shift from cold to warm conditions and again to cold conditions after the warm break (Figure S4B). An increase in EGFP signals required 14dC, and no EGFP signals were observed after 1dC in treated plants throughout the experiment (Figure S8A,B). After 14dC, the fluorescence levels remained unchanged for at least 3 days during the warm break, and the plants (14dC‐3dW) showed a priming response with a strong induction of EGFP signal after the 1‐day‐long cold treatment (14dC‐3dW‐1dC, Figure S8B). The fluorescence levels decreased after the 7‐day‐long warm break, and the priming effect disappeared (14dC‐7dW and 14dC‐7dW‐1dC, Figure S8B). *EGFP* gene expression responded quickly to temperature shifts from the initial cold conditions (1dC and 14dC) to warm breaks and again to cold conditions after the warm breaks for both 1dC and 14dC treated plants (Figure S8C,D). We observed a relatively small but statistically significant delayed effect on the downregulation of *EGFP* gene expression during the warm breaks (14dC‐3dW vs. 14dC‐7dW), as well as priming effects on its upregulation in response to the reshift to cold conditions after the warm breaks for the 14dC‐treated plants (14dC‐3dW‐1dC vs. 14dC‐7dW‐1dC, Figure S8D). Similar results were observed for the endogenous *AhgADH1* promoter at the gene expression level (Figure S8E,F).

### Introduction of long‐term cold responses into plant phenotypes

**Figure 5:**
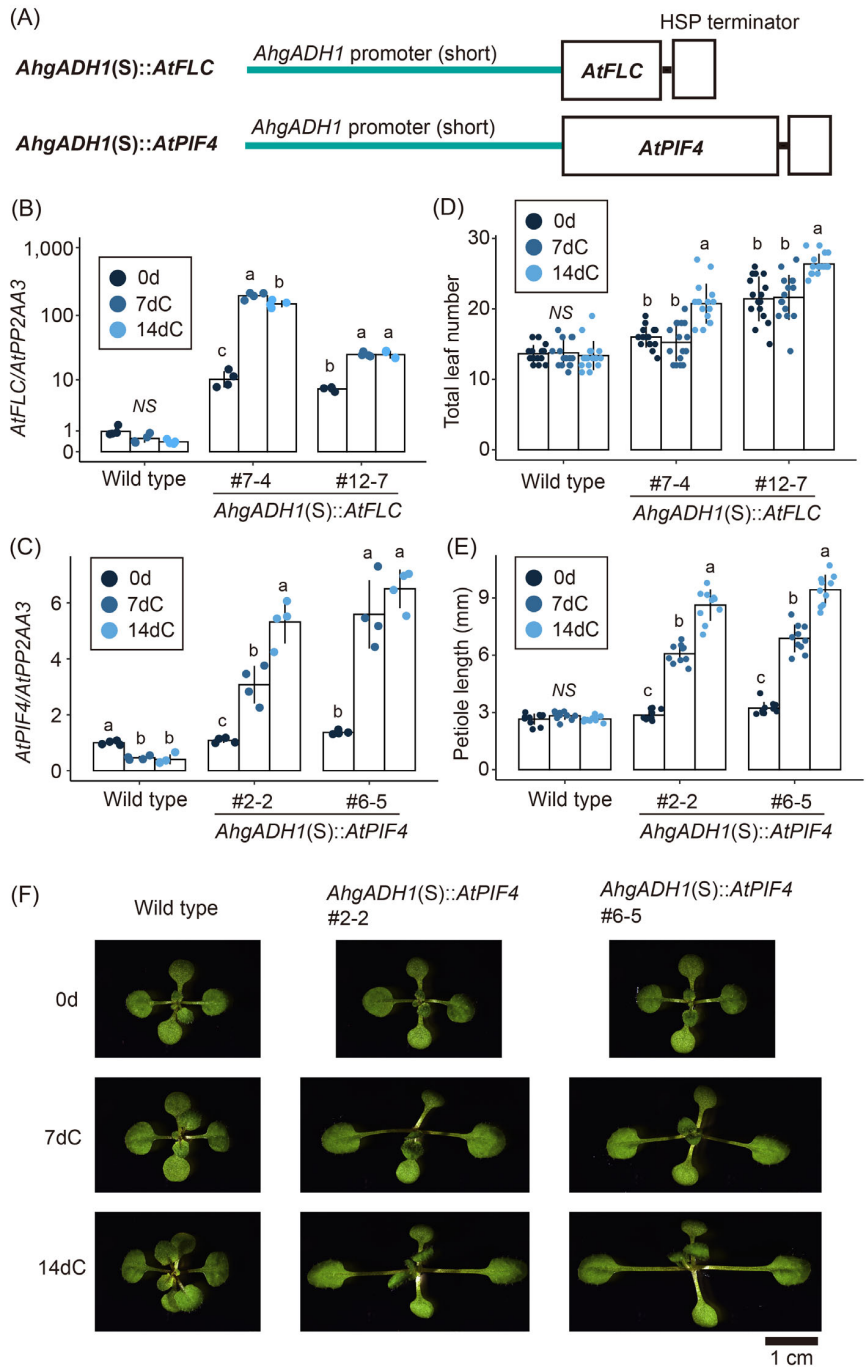
Reversed *AtFLC* and *AtPIF4* responses to long‐term cold by the WER [*AhgADH1* (S)] promoter. (A) Schematic diagrams of *AhgADH1* (S)::*AtFLC* and *AhgADH1* (S)::*AtPIF4*. The descriptions of lines and boxes are provided in the legend of Figure 3A. (B, C) *AtFLC* (B) and *AtPIF4* (C) expression levels at 0d, 7dC, and 14dC (0, 7, and 14 days after cold exposure, respectively) in the wild‐type and two representative lines of *AhgADH1* (S)::*AtFLC* (B) or *AhgADH1* (S)::*AtPIF4* (C). (B, C) Gene expression levels of both endogenous and introduced genes. Gene expression levels were quantified via RT‐qPCR and calculated relative to those of *AtPP2AA3*, with the mean value at 0d of the wild‐type = 1. Mean ± SD (*n* = 4) values are shown. (D) Flowering time responses, measured by the total number of leaves at flowering initiation, in the wild‐type and two *AhgADH1* (S)::*AtFLC* lines. Total leaf counts were performed on the main stem before flowering. (E) Petiole lengths responses in the wild‐type and two representative *AhgADH1* (S)::*AtPIF4* lines. Petiole lengths were measured for the first and second true leaves. In (D and E), mean ± SD values (*n* = 16 and 10, respectively) are shown. In (B–E), different letters indicate the significant differences among treatments (*P* < 0.05; Welch's *t*‐tests; two‐sided; diagram‐wise probability levels were adjusted using Bonferroni correction for multiple comparisons). *NS* indicates no significant difference. (F) Time‐series images of the seedlings of the wild‐type and two representative lines of *AhgADH1* (S)::*AtPIF4* (left, middle, and right columns, respectively) at 0d, 7dC, and 14dC (upper, middle, and lower columns, respectively) are shown. Scale bar: 1 cm.

The results for the promoter–GUS construct suggested that the *AhgADH1* promoter alone could activate protein accumulation in response to prolonged cold exposure. We investigated whether this promoter could be used to manipulate environmental responses to plant phenotypes. We chose two representative transcription factors that alter phenotypes in response to temperature: *FLC* and *PHYTOCHROME‐INTERACTING FACTOR 4* (*PIF4*). The repression of the former accelerates flowering in response to prolonged cold stress (Michaels & Amasino, 1999; Sung & Amasino, 2004), whereas the latter causes petiole elongation in response to exposure to warm conditions (Franklin et al., 2011; Sun et al., 2012). We introduced the *AhgADH1* (S)::*AtFLC* and *AhgADH1* (S)::*AtPIF4* constructs into *A. thaliana* to determine whether this phenotypic response could be reversed (Figure 5A). In response to cold stress, the expression of endogenous *AtFLC* and *AtPIF4* was suppressed in wild‐type plants. However, the *AhgADH1* promoter activated *AtFLC* and *AtPIF4* expression in response to cold stress (Figure 5B,C). After 2 weeks of cold exposure, the *AhgADH1* (S)::*AtFLC* lines showed delayed flowering (Figure 5D). In the *AhgADH1* (S)::*AtPIF4* line, cold exposure led to dose‐dependent effects on gene expression and petiole length (Figure 5C,E,F). Wild‐type plants showed no changes in flowering time or petiole length in response to the cold treatments (Figure 5D,E). These results suggest that the *AhgADH1* promoter alone confers WERs to plant phenotypes.

## DISCUSSION

The *AhgADH1* promoter defined in this study, the *AhgADH1* (S) promoter, was highly efficient in conferring long‐term (week‐scale) cold responsiveness to target genes, particularly at the protein level. In addition, the *AhgADH1* promoter induced H3K27me3 accumulation in the promoter region and the downstream gene body under warm conditions, whereas low levels of H3K27me3 accumulation were maintained under cold conditions. Furthermore, using the *AhgADH1* promoter, we successfully reversed thermomorphogenesis at the phenotypic level during flowering and petiole elongation by upregulating the *FLC* and *PIF4*, respectively, in response to prolonged cold stress. As the temperature fluctuates extensively under natural conditions, the promoter can provide robust control in response to long‐term seasonal temperature trends. This property can be used in practical applications to modify plants to express any selected gene in response to cold exposure for weeks.

The *AhgADH1* (S) promoter confers long‐term cold responsiveness at the protein level. Our detailed analysis indicates that the promoter is not only sufficient but also necessary for conferring WER_protein to the prolonged cold. The more proximal region of the *AhgADH1* promoter alone caused the elevation of gene expression in response to short‐term cold stress, as previously reported for the *AtADH1* promoter (Dolferus, Jacobs, et al., 1994; Kusano et al., 1995), but to confer WER_protein, it requires the upstream region covered by the *AhgADH1* (S) promoter.

Although we have identified the promoter that confers WER at the protein and phenotype levels, the link between gene expression and protein level requires further explanation. In the promoter–GUS lines, the *AhgADH1* (S) promoter conferred WER_protein and WER H3K27me3, but not WER_mRNA. One explanation is that WER_protein is an artifact through the accumulation of the stable GUS protein. However, the observed WER_protein cannot be explained by protein accumulation alone, since the GUS signals in *CaMV35S*::GUS did not show the accumulation effect (Figure 3B).

The discrepancy between the finding that WER is not seen at the gene expression level but is seen at the protein level requires a mechanism involving post‐transcriptional and translational regulation. Since the WER_protein is elicited by the presence of the *AhgADH1* promoter, the response must be triggered by a mechanism that originates from events close to the genomic promoter and locus. Potential mechanisms that cause WER_protein are those that produce transcript isoforms, such as alternative splicing, alternative TSS, and intron retention (Laloum et al., 2018). The 5′‐untranslated region of *ADH1* mRNA promotes translation in *A. thaliana*, tobacco, maize, and rice plants (Bailey‐Serres & Dawe, 1996; Mardanova et al., 2007; Satoh et al., 2004; Sugio et al., 2008); however, their role in long‐term cold response has not been tested. Another likely mechanism may involve the pathway associated with long noncoding RNAs (lncRNAs). For example, *cis*‐natural antisense RNAs, a class of lncRNAs, enhance mRNA translation from their loci (Bazin et al., 2017).

Another remaining question was whether the discrepancy between the control of gene and protein accumulation was due to the dynamics of H3K27me3 accumulation, as both H3K27me3 accumulation and protein accumulation showed WER at the transformed *AhgADH1* promoter loci. It has been reported that H3K27me3 levels affect mRNA isoform production via H3K4me3 (Pal et al., 2011) and H3K27me3 accumulation mediates lncRNA silencing (Wu et al., 2010) in mammals. The *FLC* promoter‐derived lncRNA *COLDWRAP* increases H3K27me3 levels and silences *FLC* (Kim & Sung, 2017); however, in this example, regulation occurs through the repression of gene expression. Therefore, further analysis of the *AhgADH1* promoter is necessary to identify the novel *cis*‐regulatory mechanisms affecting *ADH1* translation. At the mRNA level, the *AhgADH1* promoter maintained an initial high level of upregulation for at least 2 weeks of cold exposure, whereas the upregulation was transient when it lacked the 5′ half of the region (Figure S7). This result is suggestive to assume that the *AhgADH1* promoter contains an enhancer function involved in the non‐transient upregulation of mRNA and the WER_protein. Further experiments are required to determine whether the promoter contains an enhancer and whether H3K27me3 at the promoter region is involved in its control.

An additional point we should discuss is that the target sites of H3K27me3 accumulation differed between the endogenous (in *A. halleri*) and introduced *AhgADH1* promoters (in *A. thaliana*). When the *AhgADH1* promoter functions as an endogenous promoter in *A. halleri*, H3K27me3 accumulates in the promoter and is eliminated in response to long‐term cold stress. In contrast, when the *AhgADH1* promoter functioned as *AhgADH1*::GUS in *A. thaliana*, H3K27me3 accumulated in the GUS gene body, and promoter was maintained at low levels in response to long‐term cold exposure. ChIP‐seq analysis of *A. halleri* showed that the 3′‐side boundary of H3K27me3 at the *AhgADH1* promoter region was located near the TSS. The *AhgADH1* promoter examined in this study contained this region; therefore, the shift in H3K27me3 accumulation toward the gene body in *AhgADH1*::GUS could not be explained by local sequences at the original boundary (see Figures 1E and 2B). As the accumulation of H3K27me3 in the gene body is widespread in other genes (Nishio, Nagano, et al., 2020; Zhang et al., 2007), common mechanisms may be triggered by gene transformation. Although it was not statistically significant, there is a trend toward a short‐term cold response in H3K27me3 accumulation at the gene body for *AhgADH1*::GUS in *A. thaliana* (Figure 4B). This may be due to the different target sites of H3K27me3 for the transformed *AhgADH1*::GUS, as the transcription itself has been reported to remove H3K27me3 (Buzas et al., 2011). Therefore, further detailed comparisons between endogenous and transformed promoters are necessary to determine the mechanisms governing H3K27me3 boundaries in plant genes and their regulatory regions.

We identified the WER_phenotype in *AhgADH1* (S)::*AtFLC* and *AhgADH1* (S)::*AtPIF4* lines, and the former showed delayed flowering and the latter showed petiole elongation in response to prolonged cold. We detected WER_mRNA for *AhgADH1* (S)::*AtPIF4* in the comparison between 1‐ and 2‐week cold. *AhgADH1* (S)::*AtFLC* did not show WER_mRNA similar to *AhgADH1* (S)::GUS lines. Further study on WER_protein and WER_H3K27me3 is needed to rigorously determine whether the WER_phenotype in *AhgADH1* (S)::*AtFLC* has a similar control mechanism to *AhgADH1* (S)::GUS.

In conclusion, we identified a novel promoter that could elicit a long‐term environmental response over a week at the protein level. H3K27me3‐associated changes in the chromatin structure are likely to be involved in this long‐term response. It should also be noted that the region necessary and sufficient for the promoter to show WER is the distal region which overlaps with neighboring upstream genes. Further studies are required to determine whether this overlap with the upstream gene is related to H3K27me3 accumulation and changes in the promoter region, which have rarely been reported in plants. The discovery of a promoter that can control WER at both the protein and phenotypic levels is important for elucidating the mechanisms governing the long‐term environmental responses of plants. As the promoter exhibits the potential to control different genes, it provides a new tool for analyzing the functions of H3K27me3 and opens up new possibilities for modifying long‐term environmental responses in plants.

## MATERIALS AND METHODS

### Plants

We used *A. halleri* originating from a natural population in Taka‐cho, Hyogo Prefecture, Japan (Kudoh et al., 2018) and *A. thaliana* ecotype Columbia‐0 (Col‐0; CS70000; https://abrc.osu.edu/stocks/1). The former was used to screen candidate promoters for growth experiments and as a promoter source for plasmid construction. Col‐0 was used as the wild‐type host for transgenic lines in subsequent experiments.

### Screening of candidate promoter regions

Using monthly field transcriptome and ChIP‐seq data (Nishio, Nagano, et al., 2020), we identified promoter regions exhibiting H3K27me3 accumulation responses that negatively correlated with gene expression. We screened candidate genes that met three criteria, assuming that the promoter regions were between the TSS and 1, 2, or 3 kb upstream regions of the TSS. Genes exhibiting seasonal changes in H3K27me3 levels at the promoter (H3K27me3_pro_), TSS, and 1 kb downstream of the TSS (H3K4me3_gene_) and mRNA levels were selected based on the requirements of edgeR false discovery rate <0.05, cosinor *P*‐value <0.05, and log_2_ {(maximum reads per kilobase per million mapped reads [RPKM]) (minimum RPKM) − 1} >1, as previously reported (Nishio, Nagano, et al., 2020). Second, genes exhibiting H3K27me3_pro_ accumulation showing negative correlations with H3K4me3_gene_ accumulation and mRNA levels were selected. Third, genes exhibiting low H3K27me3 levels (maximum RPKM <2) were selected. The monthly values used in the screening were calculated through smooth spline fitting, using the smoothing spline function of the stats package in R (v.3.6.3). For correlations of H3K27me3_pro_ with H3K4me3_gene_ and mRNA, Spearman's rank correlation coefficients (*ρ*) were calculated using the cor function of the stats package in R.

### Nucleotide sequence similarity

*AhgADH1* coding sequence (1140 bp, without intron), the gene body (1728 bp, with intron) and the promoter (3326 bp) were used as a query and compared with the *AtADH1* coding sequence (1140 bp), the gene body (1708 bp) and the promoter (3326 bp), respectively, using the software, “emboss_needle” (https://www.ebi.ac.uk/jdispatcher/psa/emboss_needle). No gaps were observed in the coding sequence, and a match of 1088 bp was obtained. The Jaccard similarity of nucleotide sequence was calculated as (1088/1140) × 100 = 95.4%. In the gene body sequence, 104 bp gaps were observed, and a match of 1558 bp was obtained. The nucleotide sequence similarity was calculated as (1558/1770) × 100 = 88.0%. In the promoter sequence, 528 bp gaps were observed, and a match of 2817 bp was obtained. The nucleotide sequence similarity was calculated as (2817/3590) × 100 = 78.5%.

### Growth conditions

Seeds were surface‐sterilized and sown on 0.5% gellan gum plates containing Murashige–Skoog medium (FUJIFILM Wako Pure Chemical, Osaka, Japan) and 1% sucrose. After 3–4 days of cold stratification at 4°C, seeds were germinated in a multi‐chamber incubator (LH‐80WLED‐6CT; NK System, Osaka, Japan) set to 20°C, 8 h light/16 h dark [8 L/16D; photosynthetically active radiation (PAR), 160 μmol m^−2^ sec^−1^]. Plants were grown continuously under the same conditions for 14 and 28 days for *A. thaliana* and *A. halleri*, respectively, before the temperature treatments. In the warm treatment, the plants were maintained under the same conditions. For the cold treatment, plants were maintained at 5°C under 8 L/16D (PAR, 40 μmol m^−2^ sec^−1^) in another chamber of the same incubator.

### Plasmid construction

Cloning was performed using a kanamycin‐resistant vector plasmid (pRI201AN; TaKaRa Bio, Kusatsu, Japan), PrimeSTAR GXL DNA polymerase (TaKaRa Bio), and an In‐fusion HD cloning kit (Clontech; TaKaRa Bio). Primers used in this experiment are listed in Table S1. *AhgADH1* and *AtADH1* promoter sequences were amplified using primers designed from the genome sequences of *A. halleri* strain W302 (Briskine et al., 2017) and Col‐0, respectively. *AtFLC* and *AtPIF4* coding sequences were amplified from cDNA prepared from Col‐0. The amplified promoter fragments were cloned into the 5′‐side of GUS, and the amplified coding sequence fragments and an EGFP sequence fragment were exchanged with GUS and cloned into the 3′‐side of the *AhgADH1* promoter on the plasmid pRI201AN. *CaMV35S*::*GUS* (pRI 201AN‐GUS; TaKaRa Bio) and *CaMV35S*::*EGFP* were the control constructs.

All the constructed plasmids were introduced into Col‐0 using the floral dip method with the GV3101 strain of *Agrobacterium tumefaciens* (Clough & Bent, 1998). T1 seeds were selected on 0.5% gellan gum plates containing the Murashige–Skoog medium, 1% sucrose, and 100 μg ml^−1^ kanamycin sulfate (FUJIFILM Wako Pure Chemical). The copy numbers of the introduced genes were estimated from the segregation ratio of kanamycin resistance in the T2 generation, and each single‐copy line was selected as a representative line. Genetically stabilized T3 or T4 plants were used as representative lines.

### GUS staining and signal quantification

The GUS assay was performed as described previously (Jefferson, 1987). Briefly, seedlings were fixed in 90% acetone on ice for 15 min, washed with 0.1 M PBS buffer, and incubated in a staining solution containing 5 mm potassium ferricyanide, 5 mm potassium ferrocyanide, 0.1% Triton X‐100, 0.5 mg ml^−1^ 5‐bromo‐4‐chloro‐3‐indolyl‐β‐D‐glucuronic acid (X‐Gluc; BMS, Tokyo, Japan) at 37°C for 3 h. Stained seedlings were fixed and bleached in 70% ethanol for 1 days. To quantify the GUS signals, we calculated the ratio of GUS‐stained to unstained tissue areas. In the images of GUS‐stained seedlings, the numbers of pixels in the GUS‐stained (RGB [90, 120, and 120]) and unstained areas (RGB [255, 225, and 170]) were counted, allowing for variations ≤60. To extract pixels within the designated RGB ranges, OpenCV package v.4.6.0 (Bradski, 2000) in Python v.3.11.4 was used. No image modification was applied for all images.

### EGFP signal observation

The aerial parts of the seedlings were observed using a fluorescence stereomicroscope (M205FA; Leica Microsystems, Wetzlar, Germany). The exposure time of the images was 2 sec and 500 msec for *AhgADH1*::EGFP and *CaMV35S*::*EGFP*, respectively. No image modification was applied for all images.

### RT‐qPCR and ChIP‐qPCR

Total RNA was extracted from the aerial parts of seedlings frozen in liquid nitrogen using TRIzol reagent (Thermo Fisher Scientific, Waltham, MA, USA). As *ADH1* expression in roots is activated by hypoxic stress in gellan gum medium without cold treatment (Chung & Ferl, 1999), only the aerial parts of the seedlings were analyzed in this study. For each sample, 200 ng of total RNA was reverse‐transcribed using a High‐Capacity cDNA Reverse Transcription Kit (Thermo Fisher Scientific), and the resultant cDNA was used as a template for RT‐qPCR. The mRNA levels of the target genes were normalized to those of *PROTEIN PHOSPHATASE 2A SUBUNIT A3* (*PP2AA3*) homologues as internal controls for both *A. halleri* (Nishio et al., 2016) and *A. thaliana*. For ChIP‐qPCR, 0.1–0.5 g aerial parts of seedlings were fixed in 1% formaldehyde. The chromatin was extracted and sheared using a Q700 Sonicator (QSONICA, Newtown, CT, USA). The sheared chromatin was incubated with the following 1:150 diluted antibodies: anti‐H3K4me3 (07–473; Merck Millipore, Burlington, MA, USA), anti‐H3K27me3 (GTX50901; GeneTex, Irvine, CA, USA), and anti‐histone H3 (ab1791; Abcam, Cambridge, UK) antibodies. Complexes were precipitated using Dynabeads Protein G (10003D; Thermo Fisher Scientific). Targeted nucleotides were isolated from the beads and histones, and used as templates for ChIP‐qPCR. H3K4me3 and H3K27me3 ChIP DNA levels were expressed relative to H3 ChIP DNA levels in the same amplicon. Homologues of *ACTIN 2* (*ACT2*) and *FUSCA 3* (*FUS3*) in *A. halleri* (Nishio et al., 2016) and *A. thaliana* were used as internal controls for H3K4me3 and H3K27me3, respectively. We performed qPCR and data collection in technical duplicates using Fast SYBR Green Master Mix and QuantStudio7 (Thermo Fisher Scientific). All primers used in this study are listed in Table S1.

### Thermomorphogenesis evaluation

For the flowering time and petiole length measurements in the *AhgADH1*(S)::*AtFLC* and *AhgADH1*(S)::*AtPIF4* transfer experiments, Col‐0 and transgenic lines were grown under 16 L/8 D (PAR, 80 μmol m^−2^ sec^−1^) at 20°C using the same incubator described above. In the cold treatment, 14‐d‐old plants were grown under 16 L/8 D (PAR, 40 μmol m^−2^ sec^−1^) at 5°C. For the flowering time measurement, Col‐0 and *AhgADH1*(S)::*AtFLC* were transplanted to Jiffy‐7 (31 130 105; Jiffy Products, Rotterdam, Netherland) after the cold treatment and grown under 16 L/8 D (PAR, 80 μmol m^−2^ sec^−1^) at 20°C. The number of rosettes and cauline leaves on the main axis was scored at primary inflorescence emergence (Koornneef et al., 1991). For petiole length measurements, the first and second true leaves of Col‐0 and *AhgADH1*(S)::*AtPIF4* were measured after cold treatment using the ImageJ software (v1.53; National Institute of Health, Bethesda, MD, USA).

### Statistical analyses

Correlation analyses (Spearman's rank correlation) for promoter screening, Welch's *t*‐test (two‐sided), and Bonferroni correction were performed using the COR, t.test, and p.adjust functions in R v.4.2.2, respectively.

## AUTHOR CONTRIBUTIONS

HS and HK designed the research, HS and HN performed the research and analyzed the data, and HS and HK wrote the paper with input from HN.

## CONFLICT OF INTEREST

The authors declare no conflicts of interest for this work.

## Supporting information


**Figure S1.** Comparisons of eight putative WER promoters. (A) Monthly changes in the mRNA, H3K27me3_pro_, and H3K4me3_gene_ levels of the five genes that were not shown in Figure 1D. (B) Overlapping genes with the eight promoters. Neighboring upstream genes within 2 kb‐upstream regions from the TSS were considered to be overlapped. (C) Ratio of genes with promoters overlapping with the neighboring upstream genes (overlapping ratio) among all genes, and a histogram of overlapping base number. (D) Shared motifs among the eight promoters. (E) Shared motifs among the top three promoters with the strongest negative correlations between mRNA and H3K27me3_pro_.
**Figure S2**. Scatter plots of monthly changes in mRNA, H3K27me3_pro_, and H3K4me3_gene_ levels at the *AhgADH1* locus. Plots are shown between (A) H3K27me3_pro_ and mRNA, (B) H3K27me3_pro_ and H3K4me3_gene_, and (C) H3K4me3_gene_ and mRNA. H3K27me3_pro_ and H3K4me3_gene_ levels were calculated for the 2 kb‐upstream and 1 kb‐downstream regions from the transcription start site (TSS), respectively. Values are normalized as the maximum value = 1 for each variable. Spearman's rank correlation coefficients (ρ) are shown in the diagrams. Different colors indicate different months.
**Figure S3**. Monthly changes in the mRNA, H3K27me3_gene_, and H3K4me3_gene_ levels of *AhgADH1* and *AhgPIN6*. The levels of H3K27me3_gene_ and H3K4me3_gene_ were calculated for the entire gene body region and 1 kb region downstream of the transcription start site, respectively.
**Figure S4**. Experimental designs and time points for measurements in this study. (A) Cold treatment and (B) warm break experiments.
**Figure S5**. Comparison of endogenous and transformed *AhgADH1* promoter function. Time‐series changes in (A) *AhgADH1* and (B, C) *GUS* gene expression levels under cold (5°C for 42 days, grey lines) and warm (20°C for 14 days, black lines) conditions in (A) *Arabidopsis halleri* and (B, C) *AhgADH1* (S)::GUS lines quantified via RT‐qPCR. Expression levels were calculated relative to those of *PP2AA3*, with the mean value at 0 day = 1. Asterisks indicate the significant differences between 0 day and 1dC, 7dW and 7dC, and 14dW and 14dC (*p < 0.01; **p < 0.001; Welch's *t*‐test, two‐sided). Mean ± SD (n = 4) values are shown. All qPCR primers are listed in Table S1. (A) is identical to the left panel of Figure 2F and (B) is identical to Figure 4F, respectively.
**Figure S6**. Alignment between *ADH1* promoters of *A. halleri* and *A. thaliana*. Sequences of the *ADH1*(S) promoter region for *A. halleri* and *A. thaliana* defined in this study are shown. The sequences were aligned using the software, “emboss_needle” (https://www.ebi.ac.uk/jdispatcher/psa/emboss_needle). The *ADH1* promoter regions defined in this study and those of Dolferus, Jacobs, et al., 1994 are listed. Cold responsive motifs identified in Dolferus, Jacobs, et al., 1994 are also shown by boxes.
**Figure S7**. Comparison of *AhgADH1*(558), *AhgADH1*(660), and *AhgADH1*(S) promoters in WER to cold. Photographs of EGFP fluorescence at 0 day, 1dC, and 14dC (before the treatment, and after 1 and 14 days of cold treatment, respectively) (A). EGFP gene expression of *AhgADH1*::EGFP lines quantified via RT‐qPCR at 0 day, 1dC, and 14dC (B). In (B), expression levels were calculated relative to *AtPP2AA3*, with the mean value at 0 day = 1. Different letters indicate the significant differences between the treatments (*p* < 0.05; Welch's *t*‐tests; two‐sided; diagramwise probability levels were adjusted using Bonferroni correction for multiple comparisons). *NS* indicates no significant difference. Mean ± SD (*n* = 4) values are shown. Primers used in the qPCR are listed in Table S1.
**Figure S8**. Evaluation of priming effects after WER by warm break experiments using *AhgADH1*(S)::EGFP *A. thaliana* line and *A. halleri*. EGFP fluorescence (A, B) and EGFP gene expression (C, D) at different time points (see Figure S4B) in *AhgADH1*(S)::EGFP #13. *AhgADH1* gene expression at different time points was quantified using RT‐qPCR in *A. halleri* (E, F). In (C‐F), expression levels were calculated relative to those of *PP2AA3*, with the mean value at 0 day = 1. Effects of short‐ or long‐term cold (between 0 day and 1dC/14dC) were evaluated in the left pair of bars. Effects of warm break length (between 3dW and 7dW) were evaluated in the middle pair of bars. The priming effects in the cold response after the warm breaks (between 3dW‐1dC and 7dW‐1dC) were evaluated in the right pair of bars. Asterisks indicate significant differences (*p < 0.01; **p < 0.001; Welch's *t*‐test, two‐sided, adjusted by sequential Bonferroni correction for multiple comparisons). *NS* indicates no significant difference. Mean ± SD (*n* = 4) values are shown. Primers used in the qPCR are listed in Table S1.
**Table S1**. List of primers used in this study.

## Data Availability

Field transcriptome data from *A. halleri*, qPCR data from *A. halleri* and *A. thaliana*, and plant phenotypes from *A. thaliana* are available in the Dryad Digital Repository. https://doi.org/10.5061/dryad.5mkkwh7ch. Custom codes used in screening candidate promoter regions are available at https://github.com/hnishio/WER_promoter.git.
