# Supplementary material for: Plant ADH1 promoter acts as an H3K27me3‐associated hyper‐long cold‐responsive promoter
Source: Plant J. 2025 Jun 9;122(5):e70248. doi: 10.1111/tpj.70248 (PMC12148321; doi:10.1111/tpj.70248)
Supplement: Supplementary file 1 — Figure S1. Comparisons of eight putative WER promoters. (A) Monthly changes in the mRNA, H3K27me3pro, and H3K4me3gene levels of the five genes that were not shown in Figure 1D. (B) Overlapping genes with the eight promoters. Neighboring upstream genes within 2 kb‐upstream regions from the TSS were considered to be overlapped. (C) Ratio of genes with promoters overlapping with the neighboring upstream genes (overlapping ratio) among all genes, and a histogram of overlapping base number. (D) Shared motifs among the eight promoters. (E) Shared motifs among the top three promoters with the strongest negative correlations between mRNA and H3K27me3pro. Figure S2. Scatter plots of monthly changes in mRNA, H3K27me3pro, and H3K4me3gene levels at the AhgADH1 locus. Plots are shown between (A) H3K27me3pro and mRNA, (B) H3K27me3pro and H3K4me3gene, and (C) H3K4me3gene and mRNA. H3K27me3pro and H3K4me3gene levels were calculated for the 2 kb‐upstream and 1 kb‐downstream regions from the transcription start site (TSS), respectively. Values are normalized as the maximum value = 1 for each variable. Spearman's rank correlation coefficients (ρ) are shown in the diagrams. Different colors indicate different months. Figure S3. Monthly changes in the mRNA, H3K27me3gene, and H3K4me3gene levels of AhgADH1 and AhgPIN6. The levels of H3K27me3gene and H3K4me3gene were calculated for the entire gene body region and 1 kb region downstream of the transcription start site, respectively. Figure S4. Experimental designs and time points for measurements in this study. (A) Cold treatment and (B) warm break experiments. Figure S5. Comparison of endogenous and transformed AhgADH1 promoter function. Time‐series changes in (A) AhgADH1 and (B, C) GUS gene expression levels under cold (5°C for 42 days, grey lines) and warm (20°C for 14 days, black lines) conditions in (A) Arabidopsis halleri and (B, C) AhgADH1 (S)::GUS lines quantified via RT‐qPCR. Expression levels were calculated relative to those of P [file TPJ-122-0-s001.pdf]

(A)

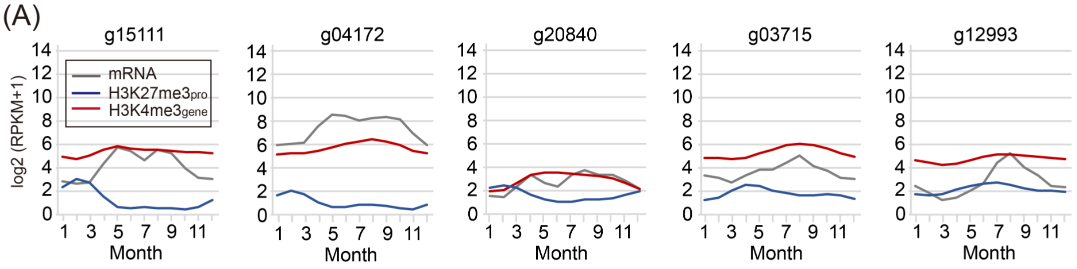

(B)

A.halleri gene code	Overlapping base number	Overlapping gene	Corresponding *A. thaliana* gene	
			AGI code	Name
g28858	46	g28859	AT2G42470	*NA*
g16364	0	-	-	-
g01268	1100	g01267	AT1G77110	*PIN6*
g15111	0	-	-	-
g04172	893	g04173	*NA*	*NA*
g20840	0	-	-	-
g03715	1035	g03716	AT1G69850	*NRT1:2*
g12993	0	-	-	-

(C)

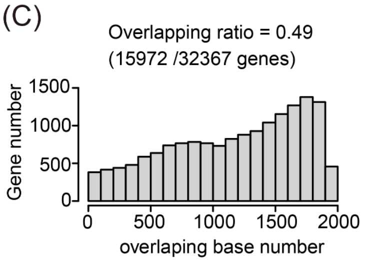

(D)

CAATTATTAATKA (E-value = 1.53e-008)

gene code	Position (Direction)	Score	pvalue	qvalue	Sequence	Motifs of ATHBs
g28858	305-317 (-)	45.2	3e-05	0.0553	TAATTATTAAAAA	*
g16364	1684-1696 (-)	52.7	5.35e-06	0.0211	TAATTATTAGTGA	*
	550-562 (+)	50.4	9.16e-06	0.0211	AAATTAATAATTA	
	557-569 (-)	50.4	9.16e-06	0.0211	AAATTAATAATTA	
	1165-1177 (+)	50.4	9.16e-06	0.0211	AAATTAATAATTA	
	1172-1184 (-)	50.4	9.16e-06	0.0211	AAATTAATAATTA	
g01268	1727-1739 (-)	68.6	1.39e-07	0.00383	CAATTATTAATGA	*
g15111	1706-1718 (+)	50.4	9.16e-06	0.0211	AAATTAATAATTA	
	1713-1725 (-)	50.4	9.16e-06	0.0211	AAATTAATAATTA	
g04172	1949-1961 (+)	51	7.85e-06	0.0211	CAATTAATAATAA	
g20840	1680-1692 (+)	54.4	3.6e-06	0.0211	CAATTATTAAATA	*
	29-41 (+)	48.5	1.4e-05	0.0275	TAATTAATAACGA	
g03715	262-274 (-)	54	3.95e-06	0.0211	AAATTATTAATGC	*
g12993	940-952 (-)	58.8	1.3e-06	0.0179	CAATTATTAACTA	*
	1386-1398 (-)	49.8	1.05e-05	0.0224	CAATTAATAAATA	
	1204-1216 (-)	40.9	8.13e-05	0.14	CAATTGATAATTA	

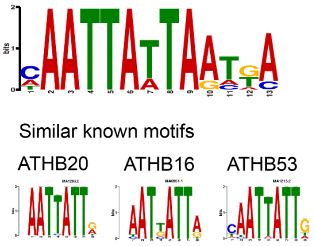

(E)

AAWTACGAASTYCKY (E-value = 3.36e-009)

gene code	Position (Direction)	Score	pvalue	qvalue	Sequence
g28858	751-765 (-)	82.9	5.05e-09	5.44e-05	AAATACGAACTCCGC
g16364	1521-1535 (-)	75.9	2.58e-08	9.25e-05	AATTACGAAGTTCGT
	1517-1531 (+)	44.7	3.4e-05	0.0915	GGGTACGAACTTCGT
g01268	1933-1947 (+)	77.1	1.93e-08	9.25e-05	AAATACGAACTTCTT

CSCAKTGTTSYYTY (E-value = 1.34e-008)

gene code	Position (Direction)	Score	pvalue	qvalue	Sequence
g28858	200-213 (-)	72	6.32e-08	0.000247	CGCAGTGTTCTTTC
	496-509 (+)	54.7	3.35e-06	0.00904	CCCATTGTTTTTTT
g16364	426-439 (-)	84.6	3.48e-09	3.76e-05	CGCATTGTTCCTTT
g1268	147-160 (-)	71.6	6.87e-08	0.000247	CCCATTGTTGCCTT
	972-985 (+)	43	5.02e-05	0.108	CCCAGGGTTATCTT
	1916-1929 (+)	41.6	6.98e-05	0.125	TCCAGTGGTGTTTT

ACGTGAAAGAH (E-value = 9.50e-007)

gene code	Position (Direction)	Score	pvalue	qvalue	Sequence
g28858	76-86 (-)	67.2	1.88e-07	0.002	ACGTGAAAGAC
g16364	1804-1814 (-)	60.4	9.04e-07	0.0048	ACGTGAAAGAA
g1268	667-677 (-)	55.8	2.62e-06	0.00928	ACGTGAAATAT
	1775-1785 (+)	40.4	9e-05	0.239	ACGTGGACGAC

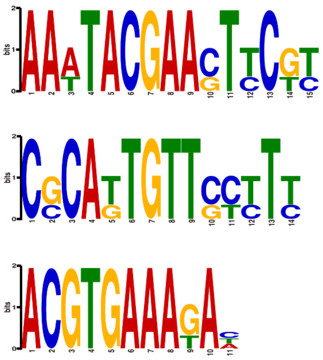

Figure S1. Comparisons of eight putative WER promoters. (A) Monthly changes in the mRNA, $H3K27me3_{pro}$, and $H3K4me3_{gene}$ levels of the five genes that were not shown in Figure 1D. (B) Overlapping genes with the eight promoters. Neighbouring upstream genes within 2 kb-upstream regions from the TSS were considered to be overlapped. (C) Ratio of genes with promoters overlapping with the neighbouring upstream genes (overlapping ratio) among all genes, and a histogram of overlapping base number. (D) Shared motifs among the eight promoters. (E) Shared motifs among the top three promoters with the strongest negative correlations between mRNA and H3K27me3pro.

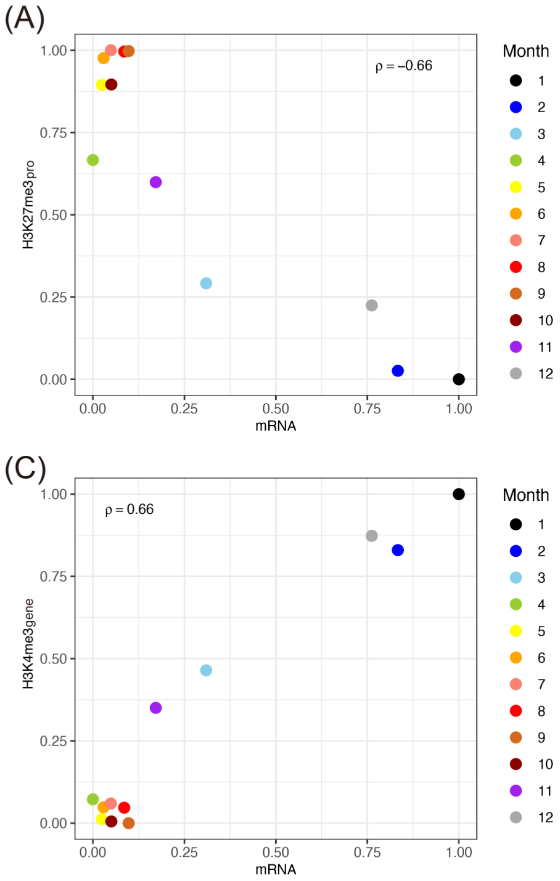

Figure S2. Scatter plots of monthly changes in mRNA, H3K27me3$_{pro}$, and H3K4me3$_{gene}$ levels at the *AhgADH1* locus. Plots are shown between (A) H3K27me3$_{pro}$ and mRNA, (B) H3K27me3$_{pro}$ and H3K4me3$_{gene}$, and (C) H3K4me3$_{gene}$ and mRNA. H3K27me3$_{pro}$ and H3K4me3$_{gene}$ levels were calculated for the 2 kb-upstream and 1 kb-downstream regions from the transcription start site (TSS), respectively. Values are normalised as the maximum value = 1 for each variable. Spearman's rank correlation coefficients (ρ) are shown in the diagrams. Different colours indicate different months.

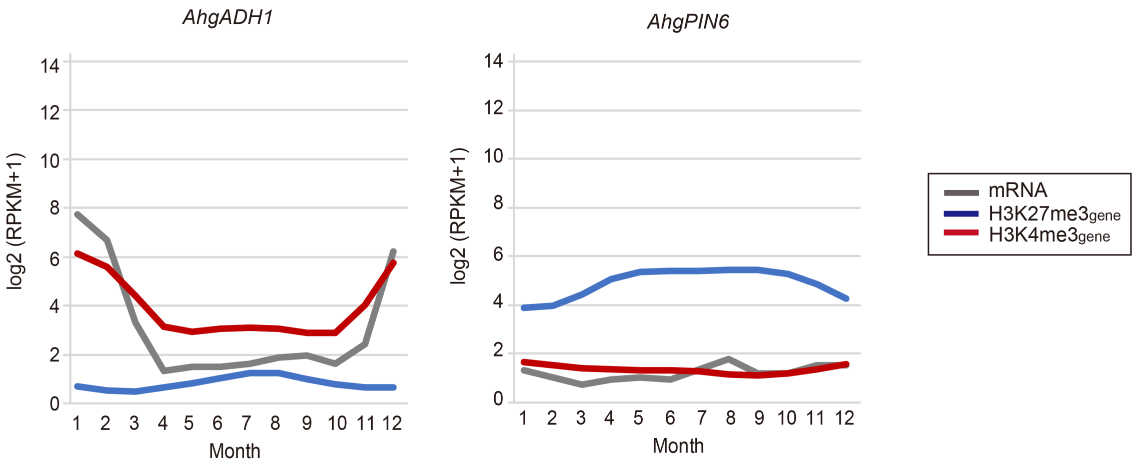

Figure S3. Monthly changes in the mRNA, H3K27me3$_{gene}$, and H3K4me3$_{gene}$ levels of *AhgADH1* and *AhgPIN6*. The levels of H3K27me3$_{gene}$ and H3K4me3$_{gene}$ were calculated for the entire gene body region and 1 kb region downstream of the transcription start site, respectively.

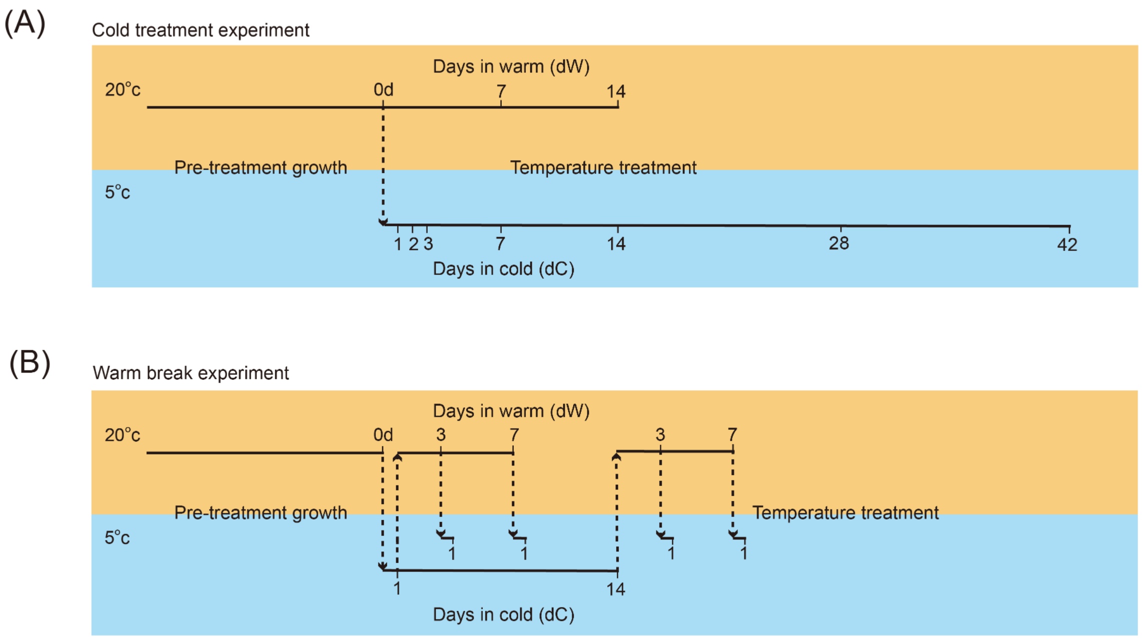

Figure S4. Experimental designs and time points for measurements in this study. (A) Cold treatment and (B) warm break experiments.

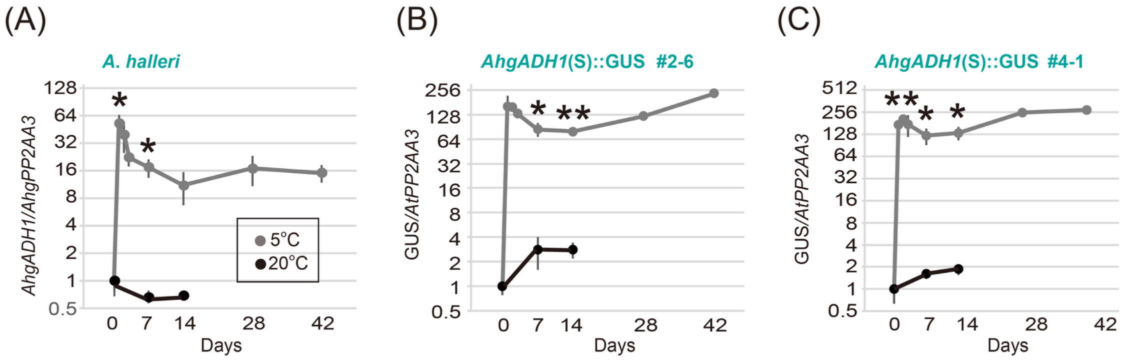

Figure S5. Comparison of endogenous and transformed *AhgADH1* promoter function. Time-series changes in (A) *AhgADH1* and (B, C) *GUS* gene expression levels under cold (5 ºC for 42 d, grey lines) and warm (20 ºC for 14 d, black lines) conditions in (A) *Arabidopsis halleri* and (B, C) *AhgADH1* (S)::GUS lines quantified via RT-qPCR. Expression levels were calculated relative to those of *PP2AA3*, with the mean value at 0 d = 1. Asterisks indicate the significant differences between 0 d and 1dC, 7dW and 7dC, and 14dW and 14dC (*$p < 0.01$; **$p < 0.001$; Welch's t-test, two-sided). Mean ± SD (n = 4) values are shown. All qPCR primers are listed in Table S1. (A) is identical to the left panel of Figure 2F and (B) is identical to Figure 4F, respectively.

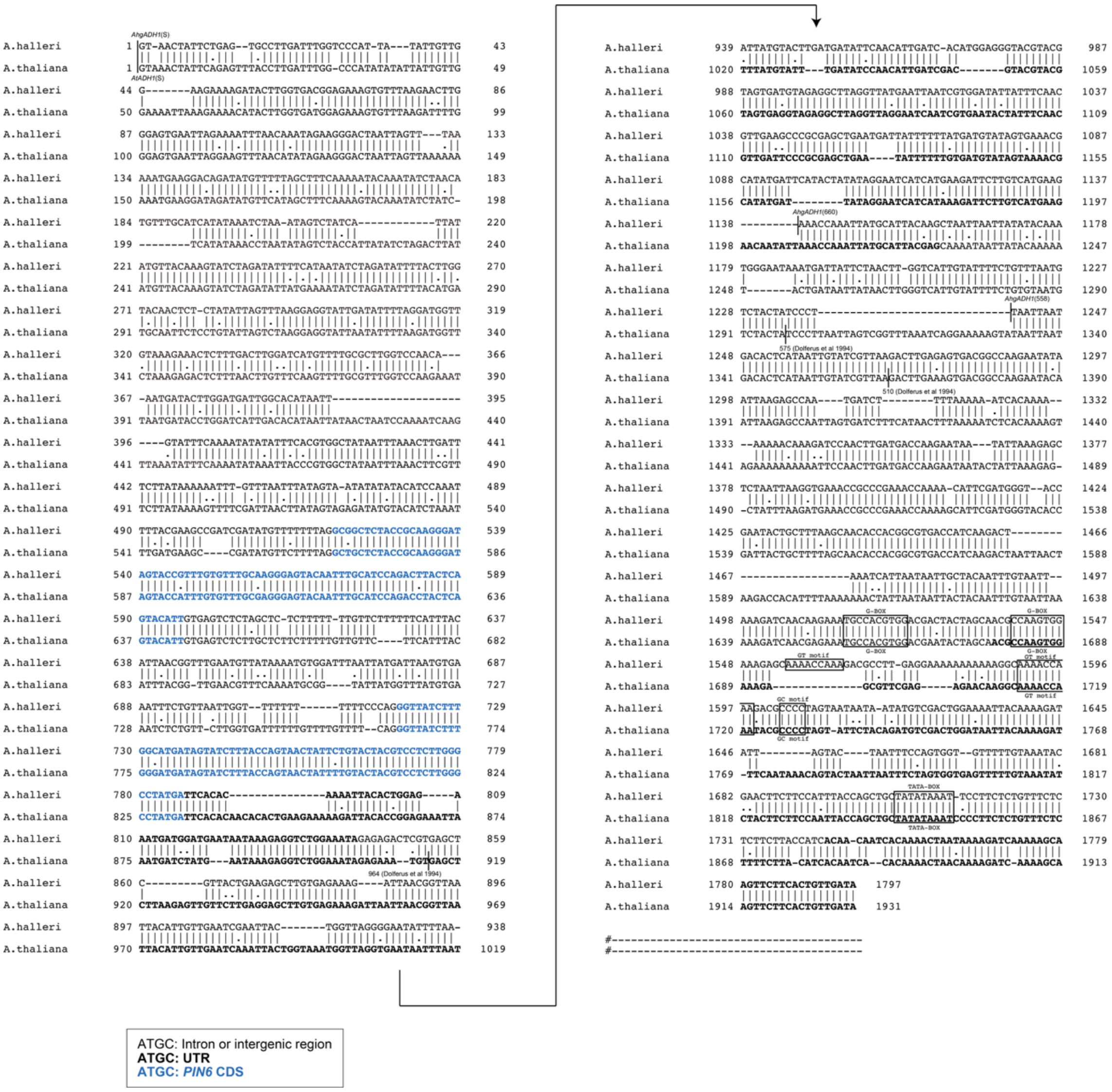

Figure S6. Alignment between *ADH1* promoters of *A. halleri* and *A. thaliana*. Sequences of the *ADH1*(S) promoter region for *A. halleri* and *A. thaliana* defined in this study are shown. The sequences were aligned using the software, 'emboss_needle' (https://www.ebi.ac.uk/jdispatcher/psa/emboss_needle). The *ADH1* promoter regions defined in this study and those of Dolferus, Jacobs, et al., 1994 are listed. Cold responsive motifs identified in Dolferus, Jacobs, et al., 1994 are also shown by boxes.

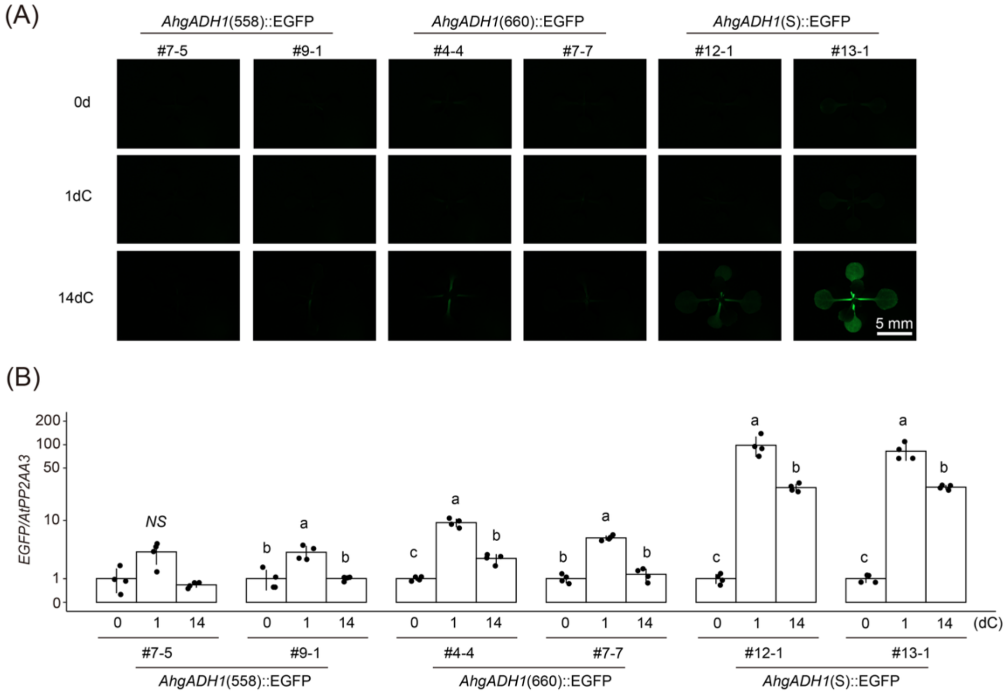

Figure S7. Comparison of *AhgADH1*(558), *AhgADH1*(660), and *AhgADH1*(S) promoters in WER to cold. Photographs of EGFP fluorescence at 0 d, 1dC, and 14dC (before the treatment, and after 1 and 14 days of cold treatment, respectively) (A). EGFP gene expression of *AhgADH1*::EGFP lines quantified via RT-qPCR at 0 d, 1dC, and 14dC (B). In (B), expression levels were calculated relative to *AtPP2AA3*, with the mean value at 0 d = 1. Different letters indicate the significant differences between the treatments ($p < 0.05$; Welch's *t*-tests; two-sided; diagram-wise probability levels were adjusted using Bonferroni correction for multiple comparisons). *NS* indicates no significant difference. Mean ± SD ($n = 4$) values are shown. Primers used in the qPCR are listed in Table S1.

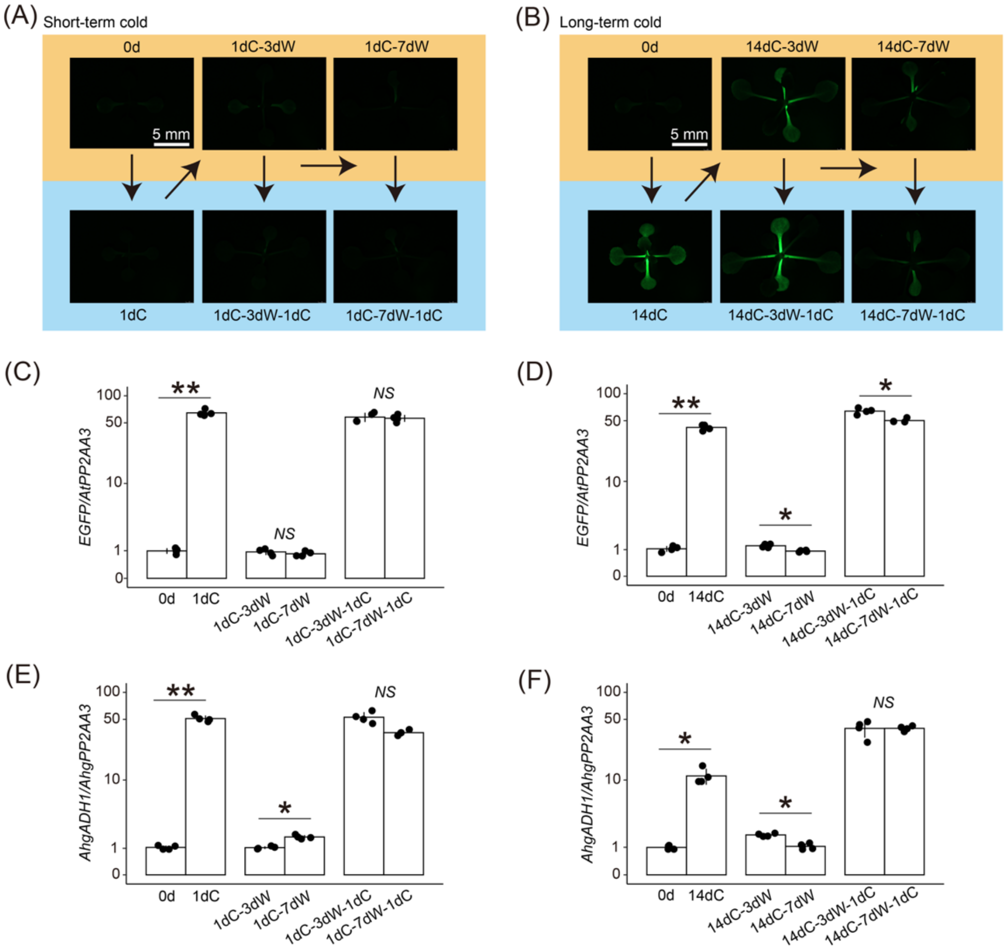

Figure S8. Evaluation of priming effects after WER by warm break experiments using *AhgADH1*(S)::EGFP *A. thaliana* line and *A. halleri*. EGFP fluorescence (A, B) and EGFP gene expression (C, D) at different time points (see Figure S4B) in *AhgADH1*(S)::EGFP #13. *AhgADH1* gene expression at different time points was quantified using RT-qPCR in *A. halleri* (E, F). In (C-F), expression levels were calculated relative to those of *PP2AA3*, with the mean value at 0 d = 1. Effects of short- or long-term cold (between 0 d and 1dC /14dC) were evaluated in the left pair of bars. Effects of warm break length (between 3dW and 7dW) were evaluated in the middle pair of bars. The priming effects in the cold response after the warm breaks (between 3dW-1dC and 7dW-1dC) were evaluated in the right pair of bars. Asterisks indicate significant differences (*$p < 0.01$; **$p < 0.001$; Welch's *t*-test, two-sided, adjusted by sequential Bonferroni correction for multiple comparisons). *NS* indicates no significant difference. Mean ± SD ($n = 4$) values are shown. Primers used in the qPCR are listed in Table S1.

Table S1. List of primers used in this study.

Experiment	Name	Sequence (5'-3')
Cloning	AhgADH1/AtADH1promoter(long)-Fw	GTACGTTTTCTTTGAAAGAT
	AhgADH1promoter(short)-Fw	GTAACTATTCTGAGTGCCTT
	AhgADH1promoter(660)-Fw	AAACCAAATTATGCATTACA
	AhgADH1promoter(558)-Fw	TAATTAATGACACTCATAAT
	AtADH1promoter(short)-Fw	GTAAACTATTCAGAGTTTACCT
	AhgADH1/AtADH1promoter-Rv	TATCAACAGTGAAGAACTTG
	AtPIF4-Fw	ATGGAACACCAAGGTTGGAG
	AtPIF4-Rv	CTAGTGGTCCAAACGAGAAC
	AtFLC-Fw	ATGGGAAGAAAAAAACTAGA
	AtFLC-Rv	CTAATTAAGTAGTGGGAGAG
RT-qPCR	AhgPP2AA3-qPCR-Fw	GTATGCACATGTTTTGCTTCCAC
	AhgPP2AA3-qPCR-Rv	CAACCAAGTCATTCTCCCTCATC
	AtPP2AA3-qPCR-Fw	GTATGCATATGTTCTGCTTCCAC
	AtPP2AA3-qPCR-Rv	CAACCAAGTCACTCTCCCTCATC
	AhgADH1/AtADH1-qPCR-Fw	GCTGGAGGGATTGTTGAGAGTG
	AtADH1-qPCR-Rv	ATCGGCAACACATGATCTCCT
	AhgADH1-qPCR-Rv	ATGGGCAACACATGATCTCCT
	AhgPIN6-qPCR-Fw	TGCCATCGTACAGGCGGCTC
	AhgPIN6-qPCR-Rv	TGCCAAAGATAACCAATGTACTGA
	AtPIN6-qPCR-Fw	TGCTATCGTACAGGCTGCTC
	AtPIN6-qPCR-Rv	TCCCAAAGATAACCAATGTACTGA
	GUS-qPCR-Fw	TACGGCAAAGTGTGGGTCAA
	GUS-qPCR-Rv	CGGCAATAACATACGGCGTG
	EGFP-qPCR-Fw	CGACCACTACCAGCAGAACA
	EGFP-qPCR-Rv	CTCGTTGGGGTCTTTGCTCA
	AtPIF4-qPCR-Fw	CTCAGATGCAGCCGATGGAG
	AtPIF4-qPCR-Rv	ACAGACGACGGTTGTTGACT
	AtFLC-qPCR-Fw	GGCTAGCCAGATGGAGAATAAT
	AtFLC-qPCR-Rv	CGGAGATTTGTCCAGCAGGT

ChIP-qPCR	AhgACT2 -Fw	GCGACCAGACAGAGAAAGAAGG
	AhgACT2 -Rv	GATGGAGAAAAGCGGAAGAAGA
	AhgFUS3 -Fw	ATTCTCAACGGAGCCCAAAC
	AhgFUS3 -Rv	AACCTCCAACGACACTCCTCTC
	AtACT2 -Fw	GCGACTTGACAGAGAAGAACA
	AtACT2 -Rv	GAAAGAAAGAGCGGAAGAAGA
	AtFUS3 -Fw	GGTGTTAACTGGAAGAAGAGAAGGA
	AtFUS3 -Rv	AGGAGGAGGGAAAGAGAGAAGG
	AhgADH1-proA-Fw	AACCAAGCTTTGTCCTACACTA
	AhgADH1-proA-Rv	CTCTGGCACTGATTCATAGTTATAATG
	AhgADH1-proB-Fw	GAGTGCCTTGATTTGGTCCC
	AhgADH1-proB-Rv	TCTTAAACACTTTCTCCGTCACCA
	AhgADH1-proC-Fw	CAAATTTTACGAAGCCGATCG
	AhgADH1-proC-Rv	CCTTGCAAACACAAACGGTA
	AhgADH1-proD-Fw	GAGAGACTCGTGAGCTCGTT
	AhgADH1-proD-Rv	ACGTACGTACCCTCCATGTG
	AhgADH1-TSS-Fw	ACGAACTTCTTCCATTTACCAGC
	AhgADH1-TSS-Rv	AATCTGTCCGGTGGTAGACA
	AhgADH1-Gene1-Fw	AAAACTTTGGAGGTTTTAGAGGTTT
	AhgADH1-Gene1-Rv	TGGCTGAAGATCAGTCACTCC
	AhgADH1-Gene2-Fw	GACATTCCCGGGGTTGTAGA
	AhgADH1-Gene2-Rv	AAGTTGGCATCAGAAGATCTAAGC
	AtADH1-proA-Fw	ACACGACGGCGTTTAGTCAA
	AtADH1-proA-Rv	AGGGATTTGGAAAGTAAAAGAGACA
	AtADH1-proB-Fw	GAGTTTACCTTGATTTGGCCC
	AtADH1-proB-Rv	CACTTTCTCCATCACCAAGTATG
	AtADH1-proC-Fw	TTAGGCTGCTCTACCGCAAG
	AtADH1-proC-Rv	CGCATTTTGAAACGTTCAACCG
	AtADH1-proD-Fw	CGTAGTGAGGTAGAGGCTTAGG
	AtADH1-proD-Rv	TATTCAGCTCGCGGGAATCAA
	AtADH1-TSS-Fw	AATGCCACGTGGACGAATACT
	AtADH1-TSS-Rv	AGGGGCGTATTTGGTTTTGC
	AtADH1-Gene1-Fw	TACCGGAGAATGTGGGGAGT
	AtADH1-Gene1-Rv	GAATCATCCCTCCTCGCTCG
	AtADH1-Gene2-Fw	CCCGGGGTTGTGGAAAAGTA

	AtADH1-Gene2-Rv	TCCGAGAATGGCACTGTGTG
	GUS-TSS-Rv	CACGGGTTGGGGTTTCTACA
	GUS-Gene1-Fw	TACGGCAAAGTGTGGGTCAA
	GUS-Gene1-Rv	CGGCAATAACATACGGCGTG
	GUS-Gene2-Fw	GATTGGGGCCAACTCCTACC
	GUS-Gene2-Rv	ATGCCATGTTCATCTGCCCA
	GUS-Gene3-Fw	TGCTGTGCCTGAACCGTTAT
	GUS-Gene3-Rv	GCTAACGTATCCACGCCGTA
	35Spro-Fw	GGACCTAACAGAACTCGCCG
	35Spro-Rv	GACAAGTGTGTCGTGCTCCA
	35Spro-TSS-Fw	AGAGAACACGGGGGACTCTA
